# Effects of Sativex^®^ Versus Placebo on Glucagon-like Peptide-1, Total Ghrelin, and Subjective Appetite in Older Adults with Poor Appetite: A Protocolized Secondary Analysis of a Double-Blind, Randomized, Placebo-Controlled Crossover Trial

**DOI:** 10.3390/nu18142274

**Published:** 2026-07-11

**Authors:** Olivia Bornæs, Rikke Lundsgaard Nielsen, Louise Westberg Strejby Christensen, Ida Klitzing Storgaard, Thomas Kallemose, Helle Gybel Juul-Larsen, Juliette Tavenier, Baker Jawad, Ove Andersen, Jens Juul Holst, Bolette Hartmann, Aino Leegaard Andersen, Ingrid Poulsen, David Peick Sonne, Morten Baltzer Houlind, Mette Merete Pedersen

**Affiliations:** 1 Department of Clinical Research, Copenhagen University Hospital Amager and Hvidovre, 2650 Hvidovre, Denmark; rikke.lundsgaard.nielsen@regionh.dk (R.L.N.); louise.westberg.strejby.christensen@regionh.dk (L.W.S.C.); ida.storgaard@sund.ku.dk (I.K.S.); thomas.kallemose@regionh.dk (T.K.); helle.gybel.juul-larsen@regionh.dk (H.G.J.-L.); juliette.tavenier@regionh.dk (J.T.); baker.jawad@regionh.dk (B.J.); ove.andersen@regionh.dk (O.A.); aino.leegaard.andersen@regionh.dk (A.L.A.); ingrid.poulsen@regionh.dk (I.P.); morten.baltzer.houlind@regionh.dk (M.B.H.); mette.merete.pedersen@regionh.dk (M.M.P.); 2 Department of Clinical Medicine, Faculty of Health and Medical Sciences, University of Copenhagen, 2200 Copenhagen, Denmark; david.peick.sonne@regionh.dk; 3 Department of Drug Design and Pharmacology, University of Copenhagen, 2100 Copenhagen, Denmark; 4 The Emergency Department, Copenhagen University Hospital Amager and Hvidovre, 2650 Hvidovre, Denmark; 5 The Novo Nordisk Foundation Center for Basic Metabolic Research, Department of Biomedical Sciences, Faculty of Health and Medical Sciences, University of Copenhagen, 2200 Copenhagen, Denmark; jjholst@sund.ku.dk (J.J.H.); bhartmann@sund.ku.dk (B.H.); 6 Department of Clinical Pharmacology, Copenhagen University Hospital Bispebjerg and Frederiksberg Hospital, 2400 Copenhagen, Denmark; 7 Department of Nephrology and Endocrinology, Copenhagen University Hospital Rigshospitalet, 2100 Copenhagen, Denmark

**Keywords:** malnutrition, poor appetite, cannabis-based medicine, Sativex^®^, glucagon-like peptide-1, ghrelin, hospital, older adults

## Abstract

Background: Poor appetite, a major contributor to malnutrition, is highly prevalent among older adults and associated with adverse clinical outcomes. Nevertheless, there are currently no widely accepted or consistently effective pharmacological treatments targeting appetite that are routinely recommended alongside nutritional interventions. Medical cannabis has been proposed as a potential appetite stimulant in older adults with poor appetite through modulation of appetite-regulating hormones such as glucagon-like peptide-1 (GLP-1) and total ghrelin, but the evidence remains limited. Aims: To investigate the effects of Sativex^®^ versus placebo on postprandial GLP-1 and total ghrelin concentrations following a single dose (8.1 mg Δ9-tetrahydrocannabinol (THC) and 7.5 mg cannabidiol (CBD)), and on subjective appetite following two separated doses, in older adults with poor appetite, and to investigate the associations between plasma concentrations of THC and its metabolites on these outcomes. Methods: This protocolized secondary analysis included 17 participants (≥65 years) with poor appetite from a double-blind, randomized, placebo-controlled crossover trial. Participants received Sativex^®^ or placebo on two separate study days. GLP-1 and total ghrelin were measured following one dose of Sativex^®^/placebo and a standardized breakfast, and subjective appetite was assessed repeatedly using visual analog scales throughout the administration of two separate doses of Sativex^®^/placebo, the consumption of a standardized breakfast, and an ad libitum lunch meal. Effects/associations were analyzed using linear mixed-effects models. Results: Compared with placebo, Sativex^®^ produced an estimated 2.38 pmol/L (95% confidence intervals (CI): −0.71 to 5.46; Bonferroni-corrected *p* = 0.274) reduction in mean GLP-1 and time-dependent changes in mean total ghrelin (Bonferroni-corrected *p* = 0.054). Compared with placebo, Sativex^®^ produced minimal changes in separate subjective appetite sensations (all Bonferroni-corrected *p* = 1.00) and a slightly lower combined appetite score (−5.2 mm; 95% CI: −24.4 to 4.0; Bonferroni-corrected *p* = 1.00). Increased plasma concentrations of THC and metabolites were associated with reduced estimates of mean GLP-1 concentrations and subjective appetite, as well as increased mean total ghrelin concentrations. However, no statistically significant differences were detected for any analyses, and estimates had wide CIs, warranting cautious interpretation. Conclusions: In conclusion, no statistically significant differences were detected between Sativex^®^ and placebo in postprandial GLP-1, total ghrelin, or subjective appetite over time in older adults with poor appetite. Similarly, no statistically significant associations were observed between plasma concentrations of THC and its metabolites and GLP-1, total ghrelin, or subjective appetite. Trial registration: ClinicalTrials.gov ID: NCT05503147; EudraCT nr.: 2021-002318-15.

## 1. Introduction

A global demographic shift toward a larger proportion of older (≥65 years) adults is expected in the coming decades, accompanied by a substantial rise in age-related diseases and conditions [1]. Malnutrition is already highly prevalent among older adults, affecting 22–75% of community-dwelling older adults and 48–91% of hospitalized older adults [2,3,4,5,6,7,8,9]. As population aging continues, the burden of malnutrition is expected to increase further. Malnutrition is associated with severe adverse consequences, including functional decline, prolonged hospital stays, reduced quality of life, and increased mortality [9,10,11,12]. Malnutrition is multifactorial in origin. However, poor appetite has been identified as one of the most important contributing factors [13,14,15,16,17,18,19,20]. Poor appetite affects approximately 20% of community-dwelling older adults and up to 65% of hospitalized individuals [13,21,22,23]. It may arise secondary to underlying disease or medication use, but it can also occur in the absence of an identifiable cause; it is often attributed to age-related physiological changes and commonly referred to as anorexia of aging [24,25,26]. Nutritional intervention studies targeting malnutrition in older adults generally report modest improvements in dietary intake and body weight [27,28,29,30]. These limited effects may be exacerbated by low adherence to the intervention, and poor appetite likely plays a central role in this challenge, as affected individuals may struggle to meet nutritional requirements despite intervention efforts [31,32].

Despite its clinical importance, there are no widely accepted or consistently effective pharmacological treatments targeting appetite that are routinely recommended alongside nutritional interventions, representing a critical gap in clinical practice [33,34,35]. Pharmacological appetite stimulants may offer a strategy to address this need, particularly in patients with low compliance with nutritional interventions due to poor appetite. However, evidence supporting the use of existing appetite-stimulating medications such as mirtazapine and megestrol acetate in older adults with poor appetite remains limited. While some studies have reported weight gain associated with megestrol acetate [24,35,36,37,38,39,40], these treatments may produce undesirable side effects, including counterproductive alterations in body composition, with weight gain primarily attributable to increased fat mass rather than lean mass [37,38,39,40].

Medical cannabis has emerged as a possible appetite-stimulating alternative, as the exogenous cannabinoid compound, Δ9-tetrahydrocannabinol (THC), may stimulate appetite via interaction with the endocannabinoid system (ECS) [41,42,43]. The ECS is a complex cellular signaling network involved in regulating several physiological processes, including appetite, digestion, and metabolism [41,44]. Age-related alterations in appetite-regulating hormones have been observed in older adults. Compared with younger individuals, older adults have been reported to have higher fasting and postprandial concentrations of the anorexigenic hormones cholecystokinin, leptin, and insulin, as well as peptide YY (PYY), alongside lower concentrations of the orexigenic hormone ghrelin [45,46,47]. Evidence from other populations indicates that cannabis exposure can modulate several of these hormones. Riggs et al. reported that smoked medical cannabis increased plasma concentrations of ghrelin and leptin and decreased PYY in men with HIV (mean age 43) [48], although food intake was not controlled. Similarly, Farokhnia et al. found that cannabis exposure (oral or smoked/vaporized) reduced plasma glucagon-like peptide-1 (GLP-1) measured 2.5 h after meal intake compared with placebo in healthy adult cannabis users (mean age 28) [49]. Together, these findings suggest that cannabis may influence hormonal pathways involved in appetite regulation. However, high-quality studies are still needed to clarify the extent and clinical relevance of these effects in older adults with poor appetite.

The aims of this study in older adults with poor appetite were to investigate (1) the effect of medical cannabis, formulated as Sativex^®^, compared with placebo on circulating GLP-1 and total ghrelin concentrations; (2) the effect of Sativex^®^ compared with placebo on subjective appetite; (3) the associations between plasma concentrations of THC and its metabolites and circulating GLP-1 and total ghrelin concentrations; and (4) the association between plasma concentrations of THC and its metabolites and subjective appetite.

## 2. Materials and Methods

### 2.1. Study Design and Setting

The present study is a protocolized secondary analysis from a double-blind, randomized (1:1), placebo-controlled crossover trial evaluating the efficacy of Sativex^®^ as an appetite stimulant in older adults with poor appetite. The trial is registered at ClinicalTrials.gov (Identifier: NCT05503147, registration: 13 April 2022) and at the European Clinical Trials Database (EudraCT nr.: 2021-002318-15, registration: 15 September 2021). The trial design, power calculation, and statistical analysis plan have been described in detail in the study protocol [50]. The exploratory analyses of THC and its metabolites addressing aims 3 and 4 were not pre-specified and were therefore not described in the published protocol. These analyses were added to the current study after it became apparent that plasma concentrations of THC and its metabolites in our study participants exhibit substantial interindividual variability, as reported by Storgaard et al. [51]. The sample size calculation was not powered for the outcomes reported in this study. Results on the primary outcome (caloric intake), safety parameters, and the pharmacokinetics of Sativex^®^ have been reported elsewhere [51,52].

In short, 73 participants were included in the trial and 17 completed the study (Figure S1). Participants were recruited from the Emergency Department (ED) at Copenhagen University Hospital, Hvidovre, Denmark, between February 2022 and December 2023. The inclusion criteria were ≥65 years of age, acutely admitted for a medical illness, a reported poor appetite defined as a score of ≤14 on the Simplified Nutritional Appetite Questionnaire (SNAQ) [53], a body mass index (BMI) ≤30 kg/m^2^, and the ability to cooperate cognitively and physically. Patients were excluded if they, among other criteria, had regular cannabis use, a terminal illness, stroke, acute myocardial infarction, or severe heart failure (New York Heart Association class III–IV) [52].

On weekdays, eligibility of newly admitted patients was assessed by a study physician. Eligible patients were approached in the ED by trained trial staff, and baseline data were collected immediately following written informed consent.

### 2.2. Study Days

After hospital discharge, each participant completed two identical study days in a randomized, crossover design. The study days were separated by a 14-day washout period. Participants received Sativex^®^ on one study day and placebo on the other (Figure S2). The allocation sequence was computer generated and managed by uninvolved staff, and both investigators and participants were blinded to the assigned treatment. The first study day occurred at least 8 days post-discharge, allowing participants to return to their habitual state. If more than 14 days elapsed between baseline data collection and the first study day, an additional screening for poor appetite using SNAQ was performed.

Study days were conducted at the Zelo Phase 1 Unit at Copenhagen University Hospital Bispebjerg, Denmark. Participants were instructed to fast for 12 h prior to each study day, with water and prescribed medication permitted. Each study day lasted ~6.5 h. A morning urine sample was collected upon arrival to screen for residual THC, whereafter participants were settled in private rooms, and a peripheral venous catheter was inserted to allow for repeated blood sampling.

### 2.3. Test Meals

#### 2.3.1. Standardized Breakfast

Participants consumed 125 mL of Nutridrink^®^ Compact (available in mocha, chocolate, vanilla, or berries). Each serving provided 1263 kilojoules (kJ), comprising 11.6 g fat, 37 g carbohydrates, and 12 g protein. The same flavor was consumed on both study days, and participants were instructed to consume the meal within 15 min.

#### 2.3.2. Standardized Homogeneous Ad Libitum Lunch

Participants received a standardized 350 g serving of a homogeneous meal consisting of either beef stew or lentil curry, both with mashed potatoes. The macronutrient composition was similar between the two options, providing 2132 kJ for the beef stew and 2220 kJ for the lentil curry, including approximately 21 g of protein per serving. The same meal was provided on both study days, and participants were instructed to consume it within 25 min [50].

### 2.4. Study Drug

Sativex^®^ is an oromucosal spray containing of 2.7 mg THC and 2.5 mg cannabidiol (CBD) per spray, along with other minor naturally occurring cannabinoids, flavonoids, terpenes, and sterols, derived from *Cannabis sativa* L. (folium cum flore) and manufactured by Jazz Pharmaceuticals plc, Dublin, Ireland.

THC—the primary appetite-stimulating component of Sativex^®^—is rapidly metabolized in the liver to the active metabolite 11-hydroxy-Δ9-tetrahydrocannabinol (11-OH-THC), and subsequently to the inactive metabolite 11-nor-9-carboxy-Δ9-tetrahydrocannabinol (THC-COOH), which reflects the extent of THC metabolism [54,55].

Placebo was prepared by the Capital Region Pharmacy of Denmark, consisting of an alcohol base with peppermint and quinine flavoring, developed to match Sativex^®^ in appearance, viscosity, and taste.

### 2.5. Administration of the Sativex^®^ or Placebo

Trained staff administered Sativex^®^ or placebo. The bottles were shaken and sprayed onto three different areas of the oromucosal surface.

- First dose: Three sprays of Sativex^®^ (total dose: 8.1 mg THC and 7.5 mg CBD) or placebo, followed by the standardized breakfast 25 min later.
- Second dose: Three sprays of Sativex^®^ (total dose: 8.1 mg THC and 7.5 mg CBD) or placebo given 245 min after the first dose, followed 25 min later by the standardized homogeneous ad libitum lunch.

After each dose, participants were instructed not to talk, swallow, or move their tongue for 15 min to minimize swallowing of Sativex^®^/placebo.

### 2.6. Blood Sampling and Biochemical Analyses of GLP-1, Total Ghrelin, and THC

#### 2.6.1. Blood Sampling

Baseline blood samples for GLP-1, total ghrelin, and THC were collected approximately 0–15 min before the first dose of Sativex^®^ or placebo. Blood samples for GLP-1 and total ghrelin were further collected at 15, 30, 45, 60, 90, 120, and 180 min after completion of the standardized breakfast, as specified in the protocol [50], whereas blood samples for THC were collected at 15, 30, 75, 90, 150, 195, 240, 330, and 360 min after administration of Sativex^®^ or placebo.

#### 2.6.2. Sample Processing and Storage

Blood samples for GLP-1 and total ghrelin were collected in EDTA tubes and kept on ice until processing. Samples were centrifuged at 4 °C and plasma was stored at −80 °C until analysis.

Blood samples for THC were kept at room temperature for ≥45 min to allow for coagulation, then centrifuged for 10 min at 2200× *g* and separated. To enhance the stability, 30 µL of ascorbic acid (1 mol L^−1^) per mL were added to the serum and subsequently frozen at −80 °C.

#### 2.6.3. GLP-1 Analysis

Samples were extracted in a final concentration of 70% ethanol before measurement of GLP-1. Plasma concentrations of total GLP-1 were measured by radioimmunoassay using antiserum no. 89390, which reacts equally with intact GLP-1 (7–36) amide and its primary metabolite GLP-1 (9–36) amide. The assay had a lower detection limit of 1 pmol/L. Intra-assay coefficients of variation were <10%. All samples were measured in duplicate [56].

#### 2.6.4. Total Ghrelin Analysis

Total ghrelin concentrations were measured using a commercially available ELISA kit (cat. No. EZGRT-89K, Millipore, Burlington, MA, USA) according to the manufacturer’s instructions. The assay had a quantification range of 50–4000 pg/mL, with intra- and inter-assay coefficients of variation ≤10%.

All GLP-1 and total ghrelin analyses were performed at the Department of Biomedical Sciences, Faculty of Health and Medical Sciences, University of Copenhagen.

#### 2.6.5. THC Analysis

Samples were analyzed at the Department of Clinical Biochemistry, North Denmark Regional Hospital, Hjørring, Denmark, for THC, 11-OH-THC, and THC-COOH using a validated ultra-high performance liquid chromatography coupled with a triple quadrupole tandem mass spectrometry assay. The lower limit of quantification was 0.25 ng/mL for all analytes, with a coefficient of variation ≤15%.

### 2.7. Subjective Appetite

Subjective appetite was pre-specified in the protocol [50] and assessed using a 100 mm visual analog scale (VAS) (0 = minimum, 100 = maximum) [57,58,59,60,61]. VAS scores were collected at baseline, and an additional nine times each study day, during which two doses of Sativex^®^/placebo was administered and both the breakfast and ad libitum lunch were consumed. Five appetite sensations were assessed, with each sensation scored on a 0–100 mm scale: satiety, desire to eat, future food intake, fullness, and hunger. The combined appetite score (0–500 mm) was calculated as desire to eat + future food intake + hunger + (100 − satiety) + (100 − fullness) [57].

### 2.8. Descriptive Variables

Information extracted from the electronic health record included age, sex, C-reactive protein (CRP), hemoglobin A1c (HbA1c), and estimated glomerular filtration rate (eGFR). Self-reported information included current smoking status, unintentional weight loss (>5% within 3 months), living condition, and alcohol consumption. Participants were additionally assessed with SNAQ, with a cut-off of ≤14, indicating poor appetite [53]; the Nutrition Risk Screening 2002 (NRS-2002), which assesses the presence or severity of malnutrition by summarizing scores A and B on a scale from 0 to 7, with a score ≥3 indicating nutritional risk [62]; and the Eating Symptom Questionnaire (ESQ), which evaluates nutrition impact symptoms such as eating difficulties, nausea, and taste disturbances [63]. ESQ symptoms are rated on a 5-point scale from 1 to 5, where 1 indicates no symptoms and 5 indicates severe symptoms. At inclusion, trial staff measured each patient’s weight and height to calculate their BMI. Body composition, including the appendicular muscle mass index (AMMI) and body fat mass, was assessed using multifrequency bioelectrical impedance analysis performed with the InBody S10 body composition analyzer, Inbody, Cerritos, CA, USA [64]. Prior to measurement, information on the timing of recent food intake, fluid intake, and urination or defecation were documented. Baseline information on disease burden, admission diagnosis, and concomitant medications are reported in Nielsen et al. 2025 [52].

### 2.9. Statistical Methods

All 17 participants were included in the analyses. For descriptive analyses, median and interquartile range (IQR) are reported for continuous variables and number with percentage for categorical variables.

Area under the curve (AUC) was calculated for GLP-1 and total ghrelin from administration of Sativex^®^ or placebo at ≈ −40 min to 180 min after breakfast ended.

The effect of Sativex^®^ versus placebo on GLP-1, total ghrelin, and subjective appetite were analyzed using all available timepoints. This was done in separate models for each outcome using linear mixed effect models with fixed effects for timepoints and treatment (Sativex^®^ vs. placebo). All models were initially fitted with an interaction between treatment (Sativex^®^ vs. placebo) and timepoints to allow for a time-dependent estimate of Sativex^®^ compared with placebo. These models were compared with models without the interaction using the likelihood ratio test (LRT). If the LRT was statistically significant, the interaction was kept (time-dependent); otherwise, it was removed from the model (not time-dependent). Regardless of whether the model was time-dependent or not, timepoints were modeled using natural cubic splines with 3–7 knots distributed according to quantiles. The number of knots was determined by visual inspection of the residual plots, as well as an LRT comparing different numbers of knots.

Baseline outcome values were also included as fixed effects. However, some models failed to converge when baseline values were included, even after model simplification. Baseline values were therefore excluded from these models. To account for repeated measures within participants across study days, the models included participant-specific random intercepts and random treatment effects. The inclusion of a random slope was evaluated, but none of the models converged; therefore, random slopes were not included. No additional residual correlation structure was specified.

Exploratory analysis investigating the associations between plasma concentrations of THC, 11-OH-THC, and THC-COOH, as well as measures of GLP-1, total ghrelin, and subjective appetite, used the same modeling approach as the treatment models (Sativex^®^ vs. placebo), with treatment replaced by THC, 11-OH-THC, or THC-COOH values in separate models. In some models, the associations for THC, 11-OH-THC, and THC-COOH were found not to be linear and modeled by a second-degree polynomial. Selection of the polynomial degree was done by the same method as the spline knot selection for timepoints. As timepoints for THC, GLP-1 and total ghrelin were not the same, only timepoints for THC measurements that could be paired with GLP-1 and total ghrelin timepoints (≤10 min between the timepoints) were included. The same approach was applied for 11-OH-THC and THC-COOH.

A summary description of all models, time dependency and variable inclusion can be found in Table S1.

For non-time-dependent models, results are presented as a single estimate, representing the average difference between Sativex^®^ and placebo, or as the effect of a 1 µg/L increase in THC, 11-OH-THC, or THC-COOH, independent of timepoints, with 95% confidence intervals (CIs) and Wald test *p*-values.

For time-dependent models, where no single estimate for the associations could be calculated, the estimated changes over the measurement period were visualized within the Sativex^®^ and placebo groups and for specific values of THC, 11-OH-THC, or THC-COOH. Additionally estimated group differences between Sativex^®^ and placebo over time were also visualized, with corresponding CIs. *p*-values for time-dependent associations were calculated by the LRT, comparing the model to an equivalent model where the exposure was removed. For the test of THC-COOH’s association with desire to eat, the reduced model excluding THC-COOH did not converge, and a *p*-value could therefore not be calculated for this association. *p*-values were corrected for multiple testing by a Bonferroni correction by multiplying the *p*-values by the number of tests performed within each composite hypothesis (two for GLP-1 and total ghrelin, and six for subjective appetite). Model assumptions regarding normal distribution were evaluated using histograms and Q-Q plots, and linearity by fitted vs. residuals plots.

A sensitivity analysis accounting for the unequal distribution of the sequence of Sativex^®^ and placebo was performed by including the sequence as a variable in all models.

No action was taken to handle missing data. However, the only missing data were values for one participant on one study day at timepoint ≈90 min for all VAS. Given the very limited extent of missing data, any potential bias is expected to be negligible.

R Version 4.5.2 was used to perform all analyses [65]. Mixed effect models were fitted using the nlme package (version 3.1-168) [66].

### 2.10. Ethics

The trial was approved by the Capital Region’s Committee on Health Research Ethics (H-21044231) and the Danish Medicines Agency (010921). Data storage and processing were authorized by the Danish Data Protection Agency under approval number P-2021-744 in October 2021. The trial was conducted between February 2022 and January 2024 and followed ICH E6 (R2) guidelines for Good Clinical Practice (GCP) and monitored by an independent monitoring unit at the University of Copenhagen. This study is reported in accordance with the CONSORT guidelines [67].

## 3. Results

The 17 participants had a median age of 78 years (IQR: 71–85), a median BMI of 19.8 (IQR: 18.3–23.4), and the majority were female (76.5%) and living alone (70.6%). The median total SNAQ score was 11 (IQR: 8–12), and poor appetite was re-assessed in 12 out of 17 participants before the study days, allowing for confirmation of their appetite status prior to participation. All participants reported eating <75% of their habitual dietary intake during the week prior to admission (Table 1).

The 17 participants who completed the study differed from the 56 who dropped out in a few baseline characteristics. Completers reported slightly more frequent daily alcohol consumption, had a slightly lower BMI, and were less likely to report consuming 0–25% of their habitual dietary intake but more likely to report consuming 25–50% of their habitual dietary intake. Participants were similar with respect to all other reported baseline characteristics. A complete overview of the baseline characteristics of participants who dropped out is provided in Table S2.

Nutrition impact symptoms, body composition, and biomarker concentrations were similar on Sativex^®^ and placebo study days (Table 2).

The AUC for GLP-1 and total ghrelin were comparable between Sativex^®^ and placebo study days (Table 3).

**Figure 1:**
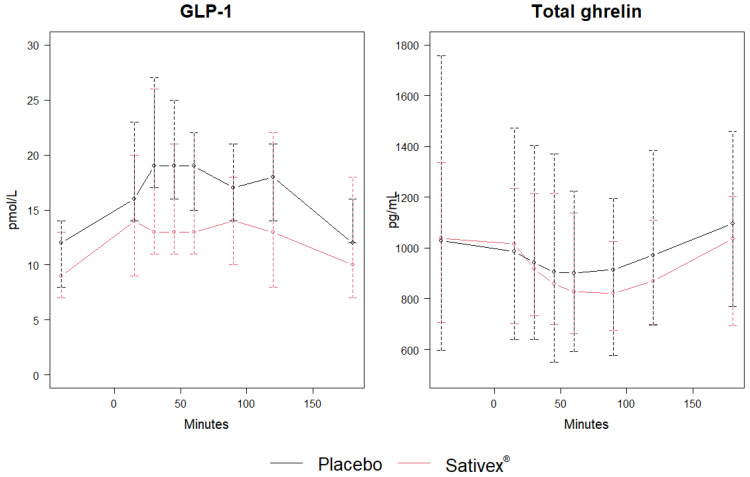
Median (interquartile range (IQR)) glucagon-like peptide-1 (GLP-1) and total ghrelin concentration time courses following Sativex^®^ and placebo administration (N = 17). Note: The first timepoint is the baseline sample at ~−40 min. Sativex^®^/placebo was administered at ~−40–25 min and 0 min is the timepoint for breakfast ending. Abbreviations: GLP-1 = glucagon-like peptide-1.

**Figure 2:**
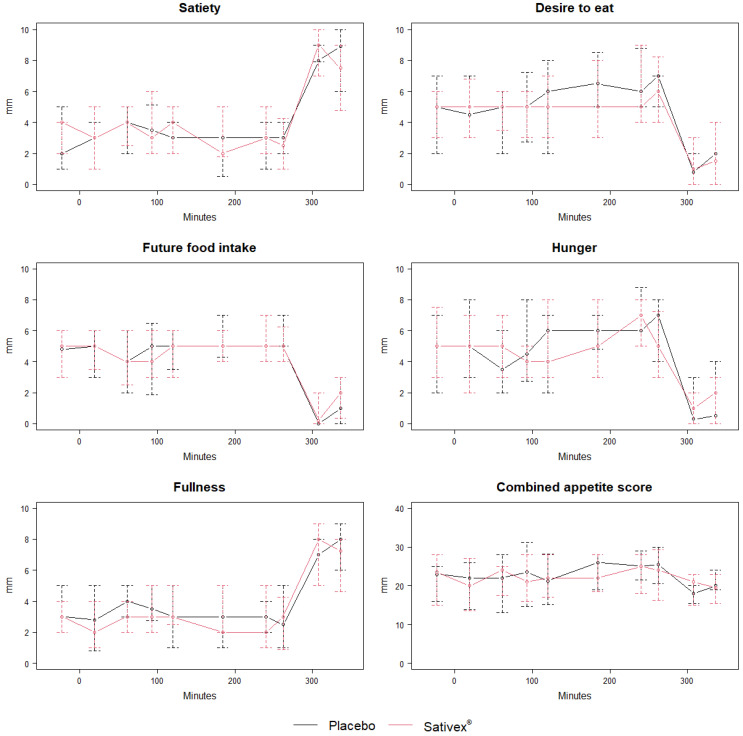
Median (interquartile range (IQR)) appetite sensation time courses following Sativex^®^ and placebo administration (N = 17). Note: The first timepoint is the baseline sample at ~−40 min. Sativex^®^ and placebo was administered at ~−25–40 min and 0 min is the timepoint for breakfast ending. Timepoints are collapsed to mean values at each timepoint. One participant had missing values for all visual analogue scales (VASs) for the placebo study day at the timepoint at ≈90 min.

GLP-1, total ghrelin, and subjective appetite time courses for Sativex^®^ and placebo are presented in Figure 1 and Figure 2.

Descriptive plots of individual THC, 11-OH-THC, and THC-COOH plasma concentrations, as well as GLP-1, total ghrelin, and subjective appetite, over time for all participants are presented in the Supplementary Materials in Figures S3–S26.

### 3.1. The Effect of Sativex^®^ on GLP-1 and Total Ghrelin Compared with Placebo

The estimates of Sativex^®^ compared with placebo was not time-dependent for GLP-1 (*p* = 0.424) and had an average reduction in mean GLP-1 concentration of 2.38 pmol/L (95% CI −0.71–5.46; Bonferroni-corrected *p* = 0.274).

**Figure 3:**
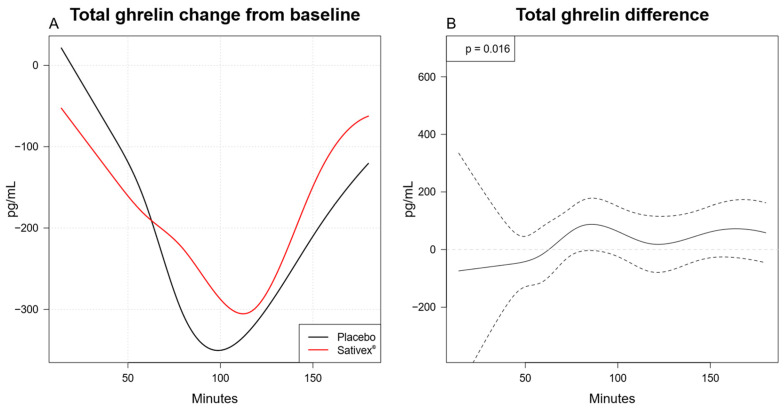
(**A**) Time-dependent changes in mean total ghrelin concentrations within Sativex^®^ and placebo groups; (**B**) differences in time-dependent changes in mean total ghrelin concentrations between Sativex^®^ and placebo groups. Note: 0 min is the timepoint for breakfast ending. For (**B**): negative values indicate larger reduction for Sativex^®^, and positive values indicate larger reduction for placebo.

In contrast, estimates of Sativex^®^ on mean total ghrelin were time-dependent (*p* = 0.016), and the estimated within-group changes over time are illustrated in Figure 3A and between-group differences with 95% CIs are illustrated in Figure 3B. Within each group, a negative mean change in total ghrelin concentrations was estimated for the majority of timepoints, with the largest reduction estimated at ~110 and ~100 min for Sativex^®^ and placebo, respectively (Figure 3A). When evaluating the between-group difference for the change, a larger reduction was initially estimated for the Sativex^®^ group until the ~65 min timepoint, and hereafter, Sativex^®^ had a smaller estimated reduction compared with placebo (Figure 3B). However, CIs at all timepoints were wide, and the difference over time for total ghrelin was not found to be statistically significant after a Bonferroni correction (*p* = 0.054).

### 3.2. The Effect of Sativex^®^ on Subjective Appetite Compared with Placebo

As group estimates were not time-dependent for any appetite sensations or the combined score (all *p* > 0.17), these are presented as non-time-dependent group effects in Table 4. For Sativex^®^, this estimated a slightly higher mean VAS score change for satiety (0.9 mm; 95% CI: −4.5 to 6.3) and slightly lower changes for the remaining appetite sensations (−2.8 to −0.7 mm). The estimate for the combined appetite score was 5.2 mm (95% CI: −24.4 to 4.0) lower for Sativex^®^ compared with placebo. However, CIs for all appetite sensations and the combined appetite score were wide and none of the differences were found to be statistically significant before (all *p* = 0.33–0.81) and after a Bonferroni correction (all *p* = 1.00).

### 3.3. The Associations Between Plasma Concentrations of THC and Its Metabolites and GLP-1, Total Ghrelin, and Subjective Appetite

Results from the association analysis between plasma concentrations of THC and its metabolites (11-OH-THC and THC-COOH) and GLP-1, total ghrelin, and subjective appetite are presented in the Supplementary Material (Table S3). In short, for non-time-dependent estimates, increasing plasma concentrations of THC, 11-OH-THC, and THC-COOH estimated lower GLP-1 (−1.23 to −0.18 pmol/L) and higher total ghrelin (1.77 to 13.50 pg/mL) concentrations. Additionally, increasing THC plasma concentrations estimated lower satiety, desire to eat, fullness, and combined appetite score (−4.50 to −0.70 mm), as well as higher hunger (0.67 mm). In contrast, higher concentrations of 11-OH-THC and THC-COOH estimated lower hunger (−0.12 to 0.04) and combined appetite score (−3.60 to −0.39). However, all estimates had wide CIs, and none were statistically significant. Only a limited number of the time-dependent effects reached statistical significance (all *p* ≤ 0.03) (Table S3), with no consistent overall pattern (Figure S27).

### 3.4. Sensitivity Analyses

The sensitivity analysis resulted in only minor changes to all estimates and did not alter the interpretation of the results. The only exception was total ghrelin, for which the *p*-value changed from 0.054 to 0.049 for the Sativex^®^/placebo analysis, thereby crossing the conventional threshold for statistical significance. However, the effect of the estimates was still minuscule.

## 4. Discussion

To our knowledge, this study is the first to evaluate the effects of medical cannabis, formulated as Sativex^®^ compared with placebo on postprandial GLP-1 and total ghrelin concentrations, as well as subjective appetite in older adults with poor appetite. No statistically significant differences were detected between Sativex^®^ and placebo in postprandial concentrations of GLP-1 and total ghrelin, as well as subjective appetite, over time in older adults with poor appetite. Similarly, no statistically significant differences were detected between plasma concentrations of THC and its metabolites and GLP-1, total ghrelin, or subjective appetite. All estimates had wide CIs, warranting cautious interpretation.

### 4.1. Key Results Compared with Previous Literature

#### 4.1.1. GLP-1 and Total Ghrelin

An estimated increase in GLP-1 concentration in both groups was seen, which is consistent with the expected postprandial response to the standardized breakfast meal. Also, a non-significant reduction in GLP-1 was observed in the Sativex^®^ group compared with placebo, which aligns with the proposed pharmacological profile of Sativex^®^ as a potential appetite stimulant, as lower GLP-1 concentrations are generally associated with increased appetite [68]. Moreover, a reduction in total ghrelin over time in both groups was observed and primarily driven by the expected postprandial response to the standardized breakfast. Estimated between-group differences over time were only consistent with a potential appetite-stimulating effect after approximately 65 min; however, these estimates also had the largest uncertainty. Overall, these findings partially align with previous studies investigating the effects of medical cannabis on GLP-1 and total ghrelin concentrations [48,49]. The estimated reduction in GLP-1 is consistent with a study by Farokhnia et al. [49]. However, direct comparison is limited by methodological differences. Farokhnia et al. included young, regular cannabis users and used combined oral and inhaled/vaporized administration, whereas our study included older adults with poor appetite and a controlled oromucosal dose of Sativex^®^. Furthermore, Farokhnia et al. did not report hormone concentration values, which limits quantitative comparisons of GLP-1 concentrations between the two studies.

Riggs et al. found significant increases in total ghrelin following smoked cannabis in men with HIV infection [48], whereas we observed non-significant variations in ghrelin over time. Differences in administration type, dosing intensity, and study duration likely contribute to these discrepancies. In their study, cannabis was administered repeatedly over five days with individualized dosing, potentially leading to higher cumulative exposure. In contrast, our study employed a controlled dose, which may have been insufficient to produce consistent effects on ghrelin. Pharmacokinetic findings from our study sample demonstrate substantial inter-individual variability, suggesting that personalized titration regimens are likely necessary to account for individual differences in response to drugs [51]. Additionally, dietary intake differed between studies and likely influenced ghrelin concentrations, as we measured hormones postprandially after a standardized meal, whereas Riggs et al. did not provide standardized meals and relied on insulin as a proxy for dietary intake [48,69].

In our analysis of THC and its metabolites, similar to the estimates for Sativex^®^, an estimated reduction in GLP-1 was observed. In contrast, estimated increases for total ghrelin were observed for increased THC, 11-OH-THC, and THC-COOH plasma concentrations, whereas estimates for the Sativex^®^ analyses were not consistent over time, possibly due to inter-individual variability in THC plasma concentrations [51] or the influence of other constituents in Sativex^®^, such as CBD, minor cannabinoids, or other phytochemicals [55]. However, higher peak THC concentrations following Sativex^®^ administration were observed in our study sample compared with findings from another study in healthy males [51,70], which may indicate that the lack of consistent effects on GLP-1 and total ghrelin is unlikely to be explained by insufficient THC concentrations alone.

Several physiological factors may contribute to the modest and variable hormonal effects observed. Sativex^®^ is intended to be absorbed from the oromucosal area, but a proportion of the dose may have been inadvertently swallowed and absorbed via the gastrointestinal tract [71,72,73], despite the participants being instructed not to swallow or to touch the oromucosal area for 15 min after administration. This may have resulted in delayed (more than a naturally occurring delay) and variable systemic THC concentrations, as observed in our study sample [51,74], potentially creating a temporal mismatch between peak drug effects and peak postprandial hormone responses. Also, the co-administration of CBD and THC through Sativex^®^ may influence the effects of THC via CYP-inhibition, as well as cannabinoid receptor modulation [51,75], although the low CBD dose in the trial product suggests minimal effect. Age-related physiological changes, such as slower gastric emptying, may additionally influence GLP-1 and total ghrelin responses [76]. Also, hormonal responses to the standardized breakfast predominate over effects of Sativex^®^ on GLP-1 and ghrelin secretion, potentially masking treatment-related changes. Fasting conditions may therefore be required to better isolate the effects of Sativex^®^ independent of meal-induced responses [60,77]. Another consideration is that we measured total rather than acylated ghrelin, with the latter being the biologically active form [78]. Consequently, the use of total ghrelin may have obscured physiologically relevant changes, although total and active ghrelin levels generally follow each other and total levels are thought to more accurately reflect rate of secretion.

#### 4.1.2. Subjective Appetite

The non-significant reduction and wide CIs in VAS appetite scores provided insufficient evidence to support Sativex^®^ as an appetite stimulant. A similar pattern was seen across most individual appetite sensations and comparable findings were observed for THC and its metabolites (OH-THC and THC-COOH). This inconclusive effect is consistent with a lack of effect seen for caloric intake in the trial [52]. The minor differences observed in subjective appetite may indicate that the VAS is not sufficiently sensitive to capture changes in subjective appetite among older adults with poor appetite. However, the VAS has been shown to correlate with subsequent food intake in both older and healthy individuals [79], suggesting that the VAS should be a useful method.

### 4.2. Strengths and Limitations

A key strength of this study is the randomized crossover design, which minimizes between-subject variability. The use of standardized fasting conditions and standardized test meals enhances internal validity, while repeated hormone measurements over ~3 h and subjective appetite assessments over ~6.5 h provide detailed insight into postprandial dynamics of appetite-related hormones and subjective appetite. Given the complexity of appetite regulation and the lack of a standard assessment method, a multidimensional approach may be preferable. A strength of this study is therefore the multimodal assessment of appetite using both appetite-related hormones, and subjective appetite scores.

However, several limitations should be acknowledged. First, the sample size was small and powered for the primary outcome of the trial, which may limit the statistical power of our analyses on secondary outcomes. This is also expressed in the wide CIs. Second, generalizability was limited by the fact that only 17 of 73 enrolled participants completed the trial, which may limit the applicability of the findings to the broader target population. Also, the study sample consisted predominantly of older women, which restricts generalizability. Third, appetite may be influenced by factors not fully controlled for in this study, including anxiety and deterioration in sensory systems (taste, smell, and sight) [25]. Additionally, SNAQ was re-assessed only if more than 14 days had elapsed between inclusion and study day 1, which introduces some uncertainty regarding appetite status immediately prior to the study days. However, as SNAQ reflects subjective appetite over a recent period rather than immediate, state-dependent appetite sensations, the impact of this uncertainty is expectedly limited. Pharmacokinetic variability represents an additional limitation. The relative contribution of oromucosal versus gastrointestinal absorption of Sativex^®^ is not fully predictable and varies between individuals [51]. Another limitation is the assessment of subjective appetite during a period with an ad libitum lunch meal, as variations in dietary intake between study days could affect appetite scores at later timepoints. Nevertheless, Nielsen et al. reported only minor differences in energy and protein intake between study days, indicating a limited influence on postprandial subjective appetite [52]. Lastly, blinding was not formally assessed, which limits confirmation of its effectiveness.

## 5. Conclusions

In conclusion, no statistically significant differences were detected between administration of medical cannabis, formulated as Sativex^®^, and placebo in postprandial GLP-1 and total ghrelin concentrations, as well as subjective appetite, over time in older adults with poor appetite. Similarly, no statistically significant associations were observed between plasma concentrations of THC and its metabolites and GLP-1, total ghrelin, or subjective appetite. Our findings suggest that the effects of Sativex^®^ on appetite warrant further investigation in studies exploring higher doses, repeated administration, or individualized titration strategies. Future clinical studies are needed to test these hypotheses and to establish the optimal dosing regimen and titration strategy.

## Figures and Tables

**Table 1:** Baseline participant characteristics (N = 17).

	All Participants
Demographics	
Age, years	78 (71–85)
Sex, female	13 (76.5%)
Living alone	12 (70.6%)
Lifestyle	
Current smoker	4 (23.5%)
Daily alcohol use	5 (29.4%)
Anthropometry	
Unintentional weight loss	6 (40.0%)
BMI, kg/m^2^	19.8 (18.3–23.4)
Appetite	
SNAQ (4–20)	11 (8–12)
Nutritional status	
NRS-2002 score A	2 (2–3)
NRS-2002 score B	1 (1–1)
Dietary intake the past week	
50–75% of habitual intake	6 (50.0%)
25–50% of habitual intake	5 (41.7%)
0–25% of habitual intake	1 (8.3%)

Note: Data are presented as median (interquartile range (IQR)) or n (%). Abbreviations: BMI = body mass index; NRS-2002 = Nutrition Risk Screening 2002; SNAQ = Simplified Nutritional Appetite Questionnaire.

**Table 2:** Sativex^®^ and placebo study day characteristics before administration (N = 17).

	Sativex^®^	Placebo
ESQ		
Appetite	1 (1–1)	1 (1–1)
Eat	1 (1–1)	1 (1–1)
Nausea	1 (1–1)	1 (1–2)
Taste	1 (1–1)	1 (1–1)
Bioelectrical impedance analysis		
AMMI, kg/m^2^	5.5 (4.9–7.3)	5.6 (5.2–6.9)
Body fat mass, %	28.6 (25.3–32.4)	26.9 (25.1–31.4)
Biomarkers		
CRP, mg/L	3.0 (1.0–4.3)	3.0 (2.0–7.5)
HbA1c, mmol/mol	37.0 (33–39)	38.0 (33–41)
eGFR, mL/min/1.73 m^2^	73.0 (42.8–84.5)	67.5 (48.5–83.3)

Note: Data are presented as median (interquartile range (IQR)); eGFR is calculated with 2009 CKD-EPI equation based on creatinine. Abbreviations: AMMI = appendicular muscle mass index; CRP = C-reactive protein; eGFR = estimated glomerular filtration rate; ESQ = Eating Symptom Questionnaire; HbA1c = hemoglobin A1c.

**Table 3:** The area under the curve (AUC) for glucagon-like peptide-1 (GLP-1) and total ghrelin (N = 17).

Outcome	Sativex^®^ AUC_−40–180_	Placebo AUC_−40–180_
GLP-1, pmol/L × min	2677 (2880–3438)	3150 (2725–3726)
Total ghrelin, pg/mL × min	186,407 (132,699–258,608)	192,490 (144,916–241,993)

Note: Administration time ≈ −40; end of breakfast = 0. AUC−40–180: The time interval for AUCs was from administration time (−40 min) to 180 min after breakfast.

**Table 4:** Between-treatment effects on non-time-dependent subjective appetite.

Outcome, mm	Estimate	Lower 95% CI	Upper 95% CI	*p*-Value	*p*-Value *
Satiety	0.9	−4.5	6.3	0.75	1.00
Desire to eat	−0.7	−6.9	5.4	0.81	1.00
Future food intake	−2.8	−8.5	2.8	0.33	1.00
Hunger	−1.5	−9.1	6.1	0.70	1.00
Fullness	−1.2	−7.5	5.1	0.72	1.00
Combined appetite score	−5.2	−24.4	4.0	0.60	1.00

Note: All estimates represent the difference between Sativex^®^ and placebo. * Bonferroni-corrected. If the corrected *p*-value resulted in a value of more than 1, a value of 1 is reported. Abbreviations: CI = confidence interval.

## Data Availability

The data supporting the findings of this study are not publicly available due to ethical restrictions protecting participant confidentiality and privacy. Access to the data may be granted by the corresponding author upon reasonable request, subject to approval by the relevant ethics committee.

## Supplementary Materials

The following supporting information can be downloaded from https://www.mdpi.com/article/10.3390/nu18142274/s1: Table S1. Model structures; Table S2. Baseline characteristics of dropouts; Table S3. The associations between a one-unit increase in THC-, 11-OH-THC-, and THC-COOH concentrations and postprandial concentrations of GLP-1 and total ghrelin, as well as subjective appetite; Figure S1. Flow diagram of patient eligibility; Figure S2. Crossover trial design; Figure S3. Time courses of individual THC and postprandial GLP-1 concentrations across 17 participants following Sativex^®^ administration; Figure S4. Time courses of individual THC and postprandial total ghrelin concentrations across 17 participants following Sativex^®^ administration; Figure S5. Time courses of individual THC concentrations and desire to eat scores across 17 participants following Sativex^®^ administration; Figure S6. Time courses of individual THC concentrations and future food intake scores across 17 participants following Sativex^®^ administration; Figure S7. Time courses of individual THC concentrations and fullness scores across 17 participants following Sativex^®^ administration; Figure S8. Time courses of individual THC concentrations and hunger scores across 17 participants following Sativex^®^ administration; Figure S9. Time courses of individual THC concentrations and satiety scores across 17 participants following Sativex^®^ administration; Figure S10. Time courses of individual THC concentrations and combined appetite scores across 17 participants following Sativex^®^ administration; Figure S11. Time courses of individual 11-OH-THC and postprandial GLP-1 concentration across 17 participants following Sativex^®^ administration; Figure S12. Time courses of individual 11-OH-THC and postprandial total ghrelin concentration across 17 participants following Sativex^®^ administration; Figure S13. Time courses of individual 11-OH-THC concentrations and desire to eat scores across 17 participants following Sativex^®^ administration; Figure S14. Time courses of individual 11-OH-THC concentrations and future food intake scores across 17 participants following Sativex^®^ administration; Figure S15. Time courses of individual 11-OH-THC concentrations and fullness scores across 17 participants following Sativex^®^ administration; Figure S16. Time courses of individual 11-OH-THC concentrations and hunger scores across 17 participants following Sativex^®^ administration; Figure S17. Time courses of individual 11-OH-THC concentrations and satiety scores across 17 participants following Sativex^®^ administration; Figure S18. Time courses of individual 11-OH-THC concentrations and combined appetite scores across 17 participants following Sativex^®^ administration; Figure S19. Time courses of individual THC-COOH and postprandial GLP-1 concentrations across 17 participants following Sativex^®^ administration; Figure S20. Time courses of individual THC-COOH and postprandial total ghrelin concentrations across 17 participants following Sativex^®^ administration; Figure S21. Time courses of individual THC-COOH concentrations and desire to eat scores across 17 participants following Sativex^®^ administration; Figure S22. Time courses of individual THC-COOH concentrations and future food intake scores across 17 participants following Sativex^®^ administration; Figure S23. Time courses of individual THC-COOH concentrations and fullness scores across 17 participants following Sativex^®^ administration; Figure S24. Time courses of individual THC-COOH concentrations and hunger scores across 17 participants following Sativex^®^ administration; Figure S25. Time courses of individual THC-COOH concentrations and satiety scores across 17 participants following Sativex^®^ administration; Figure S26. Time courses of individual THC-COOH concentrations and combined appetite scores across 17 participants following Sativex^®^ administration; Figure S27. Time-dependent changes for THC, 11-OH-THC, and THC-COOH.

## Abbreviations

The following abbreviations are used in this manuscript:

GLP-1	Glucagon-like peptide-1
THC	Δ9-tetrahydrocannabinol
CBD	Cannabidiol
CI	Confidence interval
ECS	Endocannabinoid system
PYY	Peptide YY
ED	Emergency Department
SNAQ	Simplified Nutritional Appetite Questionnaire
BMI	Body mass index
kJ	Kilojoule
11-OH-THC	11-hydroxy-Δ9-tetrahydrocannabinol
THC-COOH	11-nor-9-carboxy-Δ9-tetrahydrocannabinol
VAS	Visual analog scale
CRP	C-reactive protein
HbA1c	Hemoglobin A1c
eGFR	Estimated glomerular filtration rate
NRS-2002	Nutrition Risk Screening 2002
ESQ	Eating Symptom Questionnaire
AMMI	Appendicular muscle mass index
IQR	Interquartile range
AUC	Area under the curve
LRT	Likelihood ratio test
