# Supplementary material for: Effects of Sativex® Versus Placebo on Glucagon-like Peptide-1, Total Ghrelin, and Subjective Appetite in Older Adults with Poor Appetite: A Protocolized Secondary Analysis of a Double-Blind, Randomized, Placebo-Controlled Crossover Trial
Source: Nutrients. 2026 Jul 11;18(14):2274. doi: 10.3390/nu18142274 (PMC13414925; doi:10.3390/nu18142274)

Table S1. Model structures

Exposure	Outcome	Time dependent	Included variables
Sativex®/Placebo	GLP-1	No	Sativex®/Placebo + baseline GLP-1 + time points (spline 5 knots)
	Total ghrelin	Yes	Sativex®/Placebo + baseline total ghrelin + time points (spline 5 knots)
	Satiety	No	Sativex®/placebo + baseline Satiety + time points (spline 5 knots)
	Desire to eat	No	Sativex®/placebo + baseline Desire to eat + time points (spline 6 knots)
	Future food intake	No	Sativex®/placebo + baseline Future food intake + time points (spline 5 knots)
	Hunger	No	Sativex®/placebo + baseline Hunger + time points (spline 6 knots)
	Fullness	No	Sativex®/placebo + baseline Fullness + time points (spline 5 knots)
	Combined appetite score	No	Sativex®/placebo + baseline combined appetite score + time points (spline 6 knots)
THC	GLP-1	No	THC + baseline GLP-1 + time points (spline 4 knots)
	Total ghrelin	No	THC + baseline total ghrelin + time points (spline 3 knots)
	Satiety	No	THC + baseline Satiety + time points (spline 5 knots)
	Desire to eat	No	THC + baseline Desire to eat + time points (spline 5 knots)
	Future food intake	Yes	THC + baseline Future food intake + time points (spline 5 knots)
	Hunger	No	THC + baseline Hunger + time points (spline 7 knots)
	Fullness	No	THC + baseline Fullness + time points (spline 6 knots)
	Combined appetite score	No	THC + baseline combined appetite score + time points (spline 5 knots)
11-OH-THC	GLP-1	No	11-OH-THC + time points (spline 6 knots)
	Total ghrelin	No	11-OH-THC + baseline total ghrelin + time points (spline 3 knots)
	Satiety	Yes	11-OH-THC (2. degree polynomial) + baseline Satiety + time points (spline 5 knots)
	Desire to eat	Yes	11-OH-THC + baseline Desire to eat + time points (spline 5 knots)
	Future food intake	Yes	11-OH-THC + baseline Future food intake + time points (spline 5 knots)
	Hunger	No	11-OH-THC + baseline Hunger + time points (spline 5 knots)
	Fullness	Yes	11-OH-THC + baseline Fullness + time points (spline 5 knots)
	Combined appetite score	No	11-OH-THC + baseline combined appetite score + time points (spline 5 knots)
THC-COOH	GLP-1	No	THC-COOH + baseline GLP-1 + time points (spline 4 knots)
	Total ghrelin	No	THC-COOH + baseline total ghrelin + time points (spline 3 knots)
	Satiety	Yes	THC-COOH + baseline Satiety + time points (spline 5 knots)
	Desire to eat	Yes	THC-COOH + baseline Desire to eat + time points (spline 5 knots)
	Future food intake	Yes	THC-COOH + baseline Future food intake + time points (spline 5 knots)
	Hunger	No	THC-COOH + baseline Hunger + time points (spline 5 knots)
	Fullness	Yes	THC-COOH (2. degree polynomial) + baseline Fullness + time points (spline 5 knots)
	Combined appetite score	No	THC-COOH + baseline combined appetite score + time points (spline 5 knots)

Table S2. Baseline characteristics of dropouts

	Dropouts (N = 56)
Demographics	
Age, years	79 (72 : 83.25)
Sex, female	33 (58.9%)
Living alone	38 (67.9%)
Lifestyle	
Current smoker	16 (29.6%)
Daily alcohol use	6 (10.7%)
Anthropometry	
Unintentional weight loss	9 (17%)
BMI, kg/m^2	23.5 (20.5 : 25.8)
Appetite	
SNAQ (4-20)	12.5 (10.75 : 14)
Nutritional status	
NRS-2002 score A	2 (1 : 2)
NRS-2002 score B	1 (1 : 1)
Dietary intake the past week	
50–75% of habitual intake	17 (47.2%)
25–50% of habitual intake	12 (33.3%)
0–25% of habitual intake	7 (19.4%)

Table S3. The associations between a one-unit increase in THC-, 11-OH-THC-, and THC-COOH-concentrations and postprandial concentrations of GLP-1 and total ghrelin, and subjective appetite

**Outcome**	**Exposure,** µg/L	**Estimate**	**Lower 95% CI**	**Upper 95% CI**	**p-value**	**p-value***
GLP-1, pmol/l	THC	-1.23	-2.29	-0.17	0.026	0.153
Total ghrelin, pg/mL	THC	13.50	-5.20	32.10	0.164	0.981
Satiety, mm	THC	-0.70	-2.60	1.20	0.475	1.000
Desire to eat, mm	THC	-1.43	-3.70	0.82	0.218	1.000
Future food intake, mm	THC				0.004	0.077
Hunger, mm	THC	0.67	-1.20	2.60	0.497	1.000
Fullness, mm	THC	-1.50	-3.30	0.34	0.117	1.000
Combined appetite score, mm	THC	-4.50	-9.60	0.54	0.085	1.000
GLP-1, pmol/l	11-OH-THC	-0.54	-1.21	0.13	0.123	0.740
Total ghrelin, pg/mL	11-OH-THC	6.81	-5.90	19.52	0.300	1.000
Satiety, mm	11-OH-THC				0.002	0.030
Future food intake, mm	11-OH-THC				<0.001	0.006
Hunger, mm	11-OH-THC	-0.12	-1.60	1.30	0.871	1.000
Fullness, mm	11-OH-THC				<0.001	0.006
Combined appetite score, mm	11-OH-THC	-3.60	-7.50	0.27	0.073	1.000
GLP-1, pmol/l	THC-COOH	-0.18	-0.33	-0.03	0.019	0.116
Total ghrelin, pg/mL	THC-COOH	1.77	-1.06	4.61	0.226	1.000
Satiety, mm	THC-COOH				0.067	1.000
Desire to eat, mm	THC-COOH				0.046	0.833
Future food intake, mm	THC-COOH				0.002	0.029
Hunger, mm	THC-COOH	-0.04	-0.33	0.25	0.797	1.000
Fullness, mm	THC-COOH				<0.001	0.005
Combined appetite score, mm	THC-COOH	-0.39	-1.20	0.38	0.326	1.000

Note: The "desire to eat"-model for 11-OH-THC could not be assessed due to non-convergence of the comparison model; *corrected for multiple testing; blank field = the model is time dependent.

Abbreviations: THC = delta-9-tetrahydrocannabinol; 11-OH-THC = 11-hydroxy-Δ9-tetrahydrocannabinol; THC-COOH = 11-nor-9-carboxy-Δ9-tetrahydrocannabinol

Figure S1. Flow diagram of patient eligibility

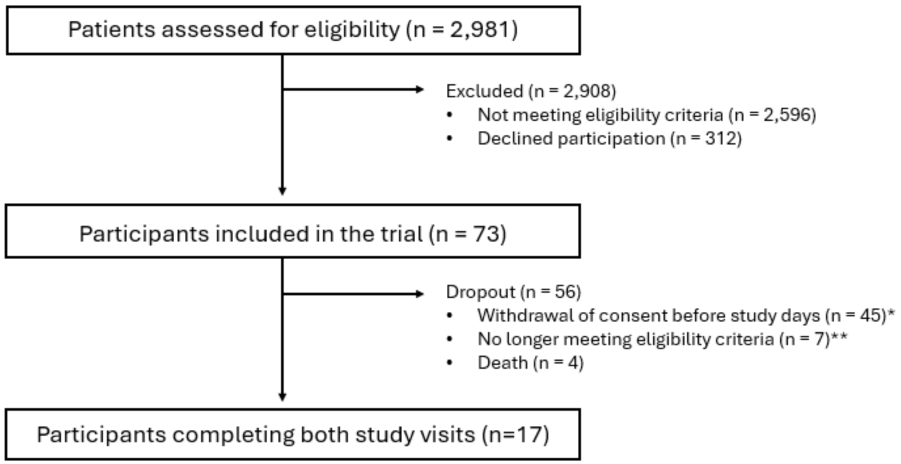

Note: All participants who discontinued the study dropped out prior to study day 1 and therefore, no attrition occurred between the two study days; *Reasons for withdrawal of consent before study days: too tired to participate (n = 25), not interested in the study (n = 7), could not be contacted (n = 4), illness (n = 3), found the study too demanding (n = 1), other reasons (n = 5); **criteria no longer met: SNAQ ≤14 (n = 5) and no cancer diagnosis (n = 2).

Figure S2. Crossover trial design

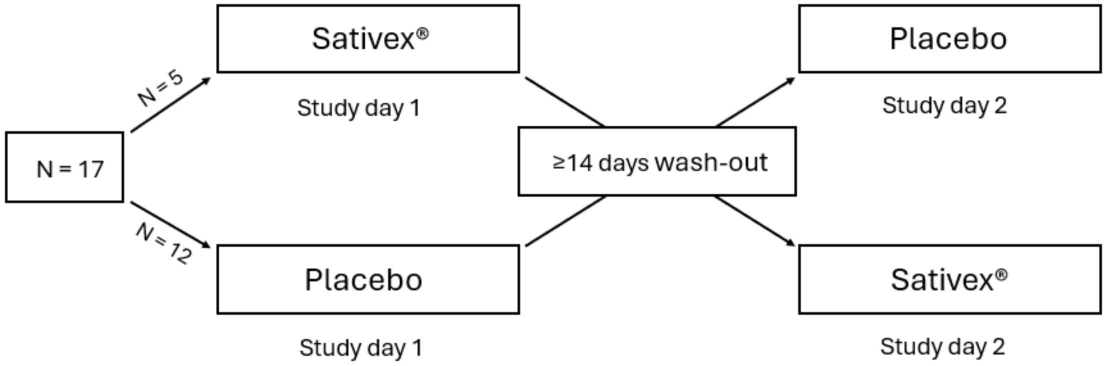

Figure S3. Time courses of individual THC and postprandial GLP-1 concentrations across 17 participants following Sativex® administration

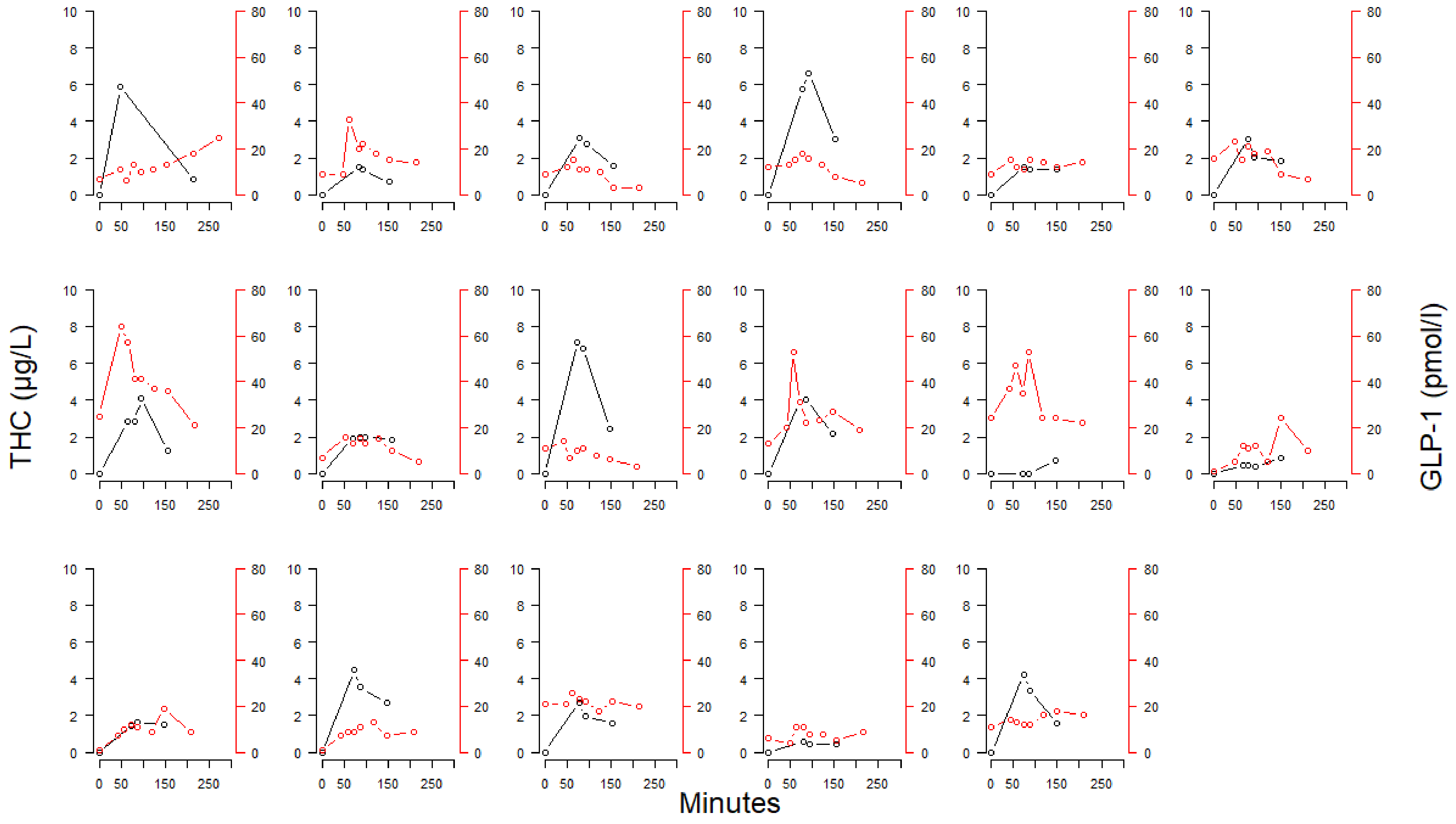

Figure S4. Time courses of individual THC and postprandial total ghrelin concentrations across 17 participants following Sativex® administration

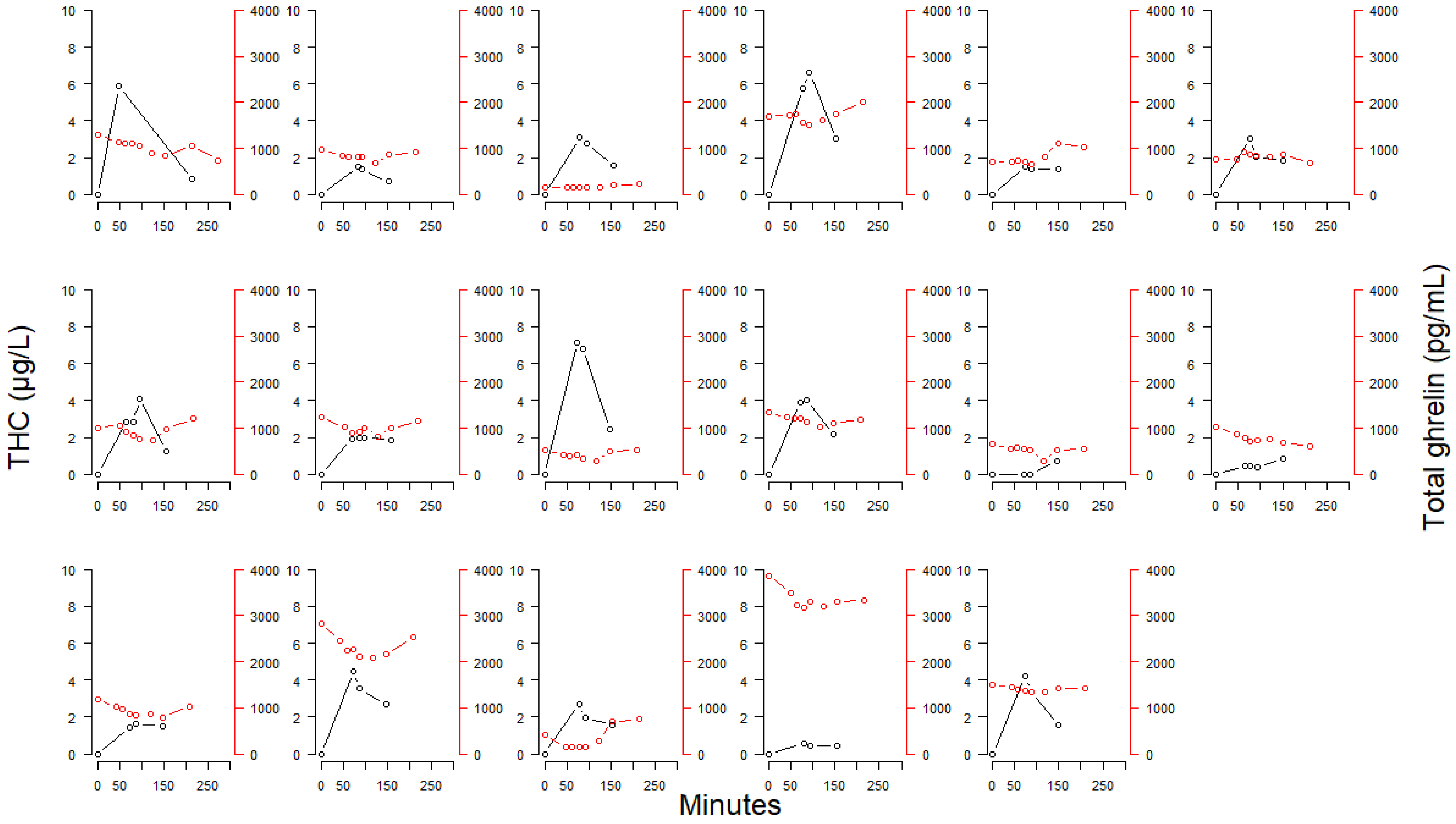

Figure S5. Time courses of individual THC concentrations and desire to eat scores across 17 participants following Sativex® administration

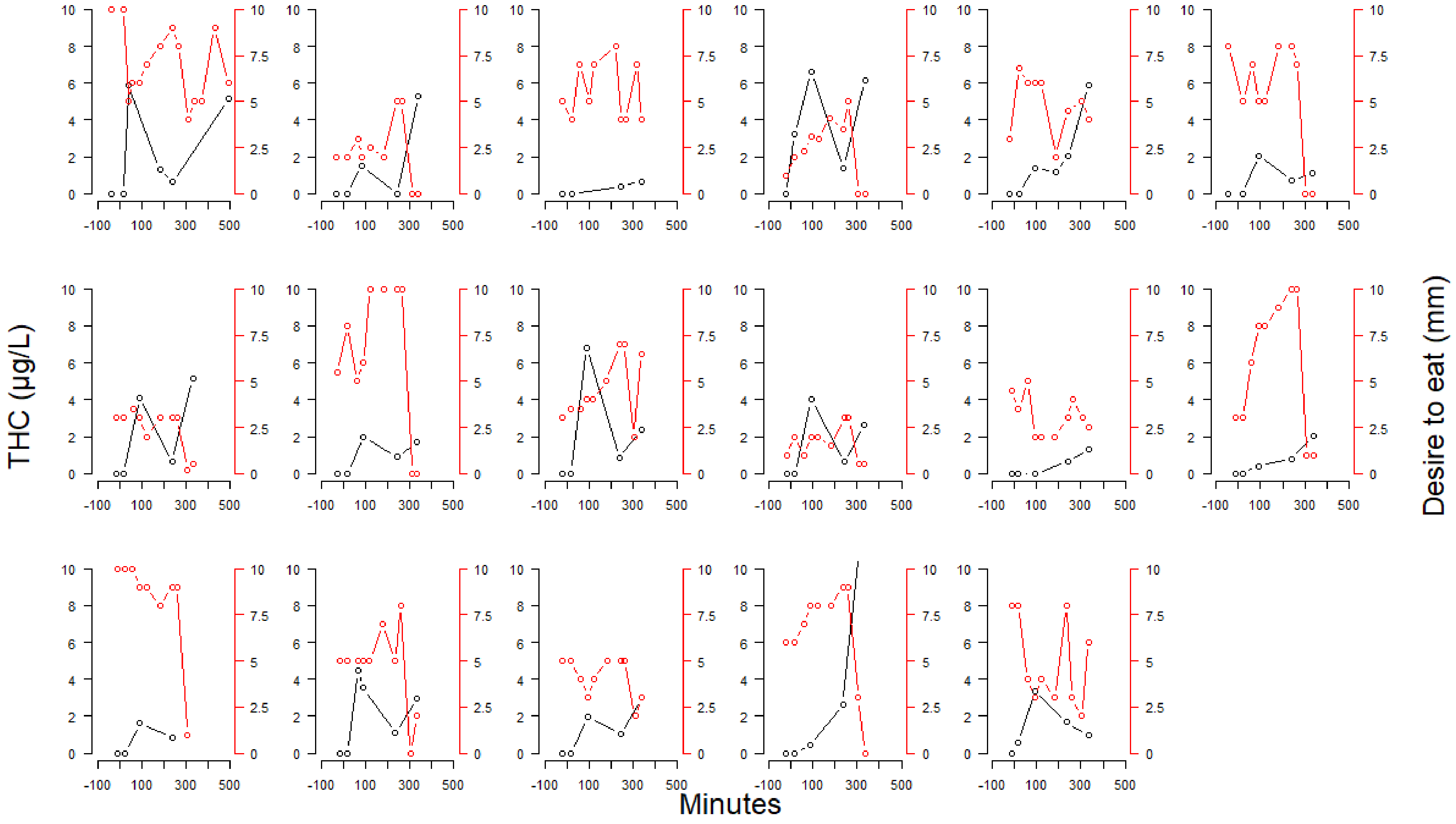

Figure S6. Time courses of individual THC concentrations and future food intake scores across 17 participants following Sativex® administration

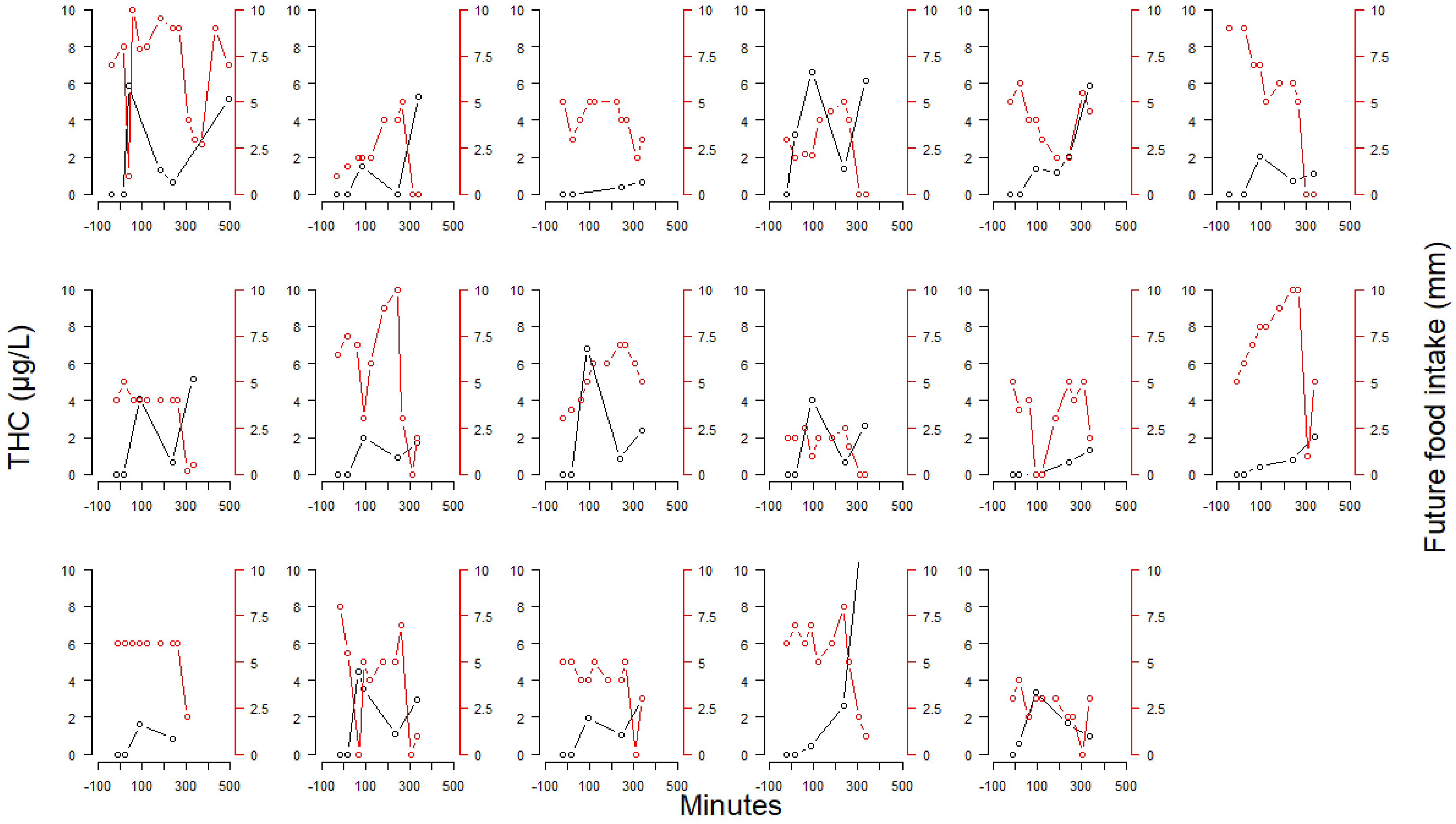

Figure S7. Time courses of individual THC concentrations and fullness scores across 17 participants following Sativex® administration

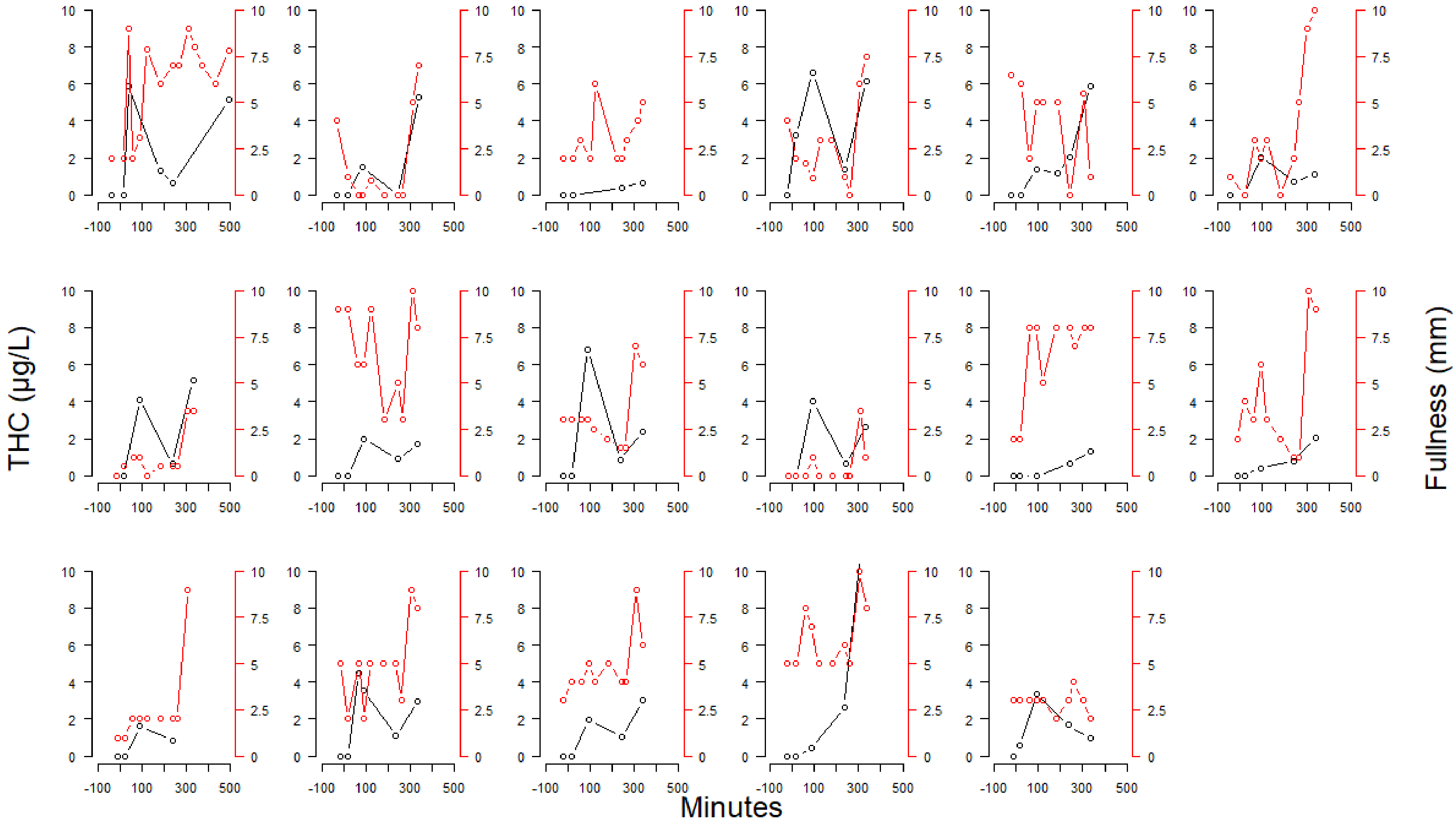

Figure S8. Time courses of individual THC concentrations and hunger scores across 17 participants following Sativex® administration

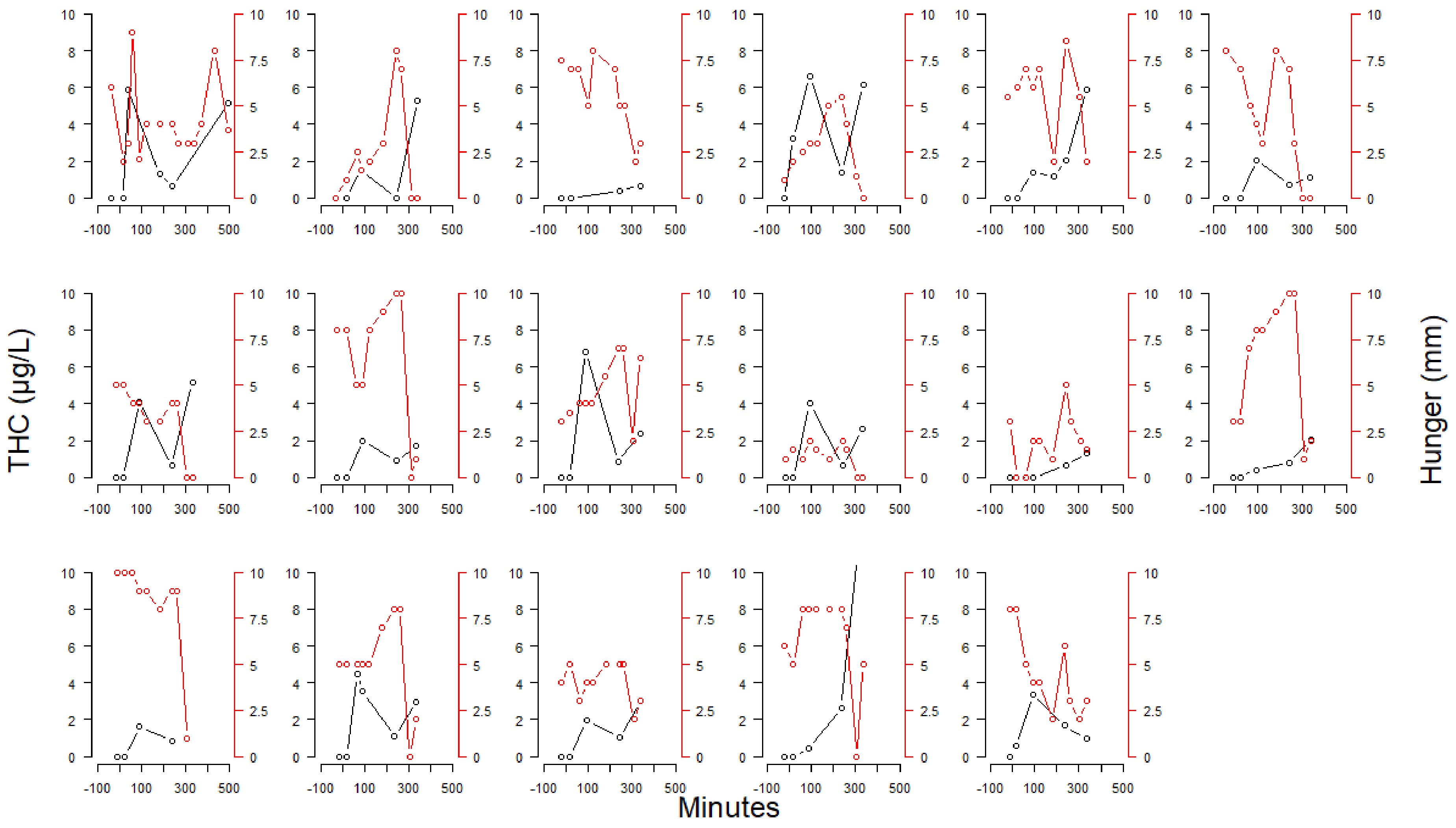

Figure S9. Time courses of individual THC concentrations and satiety scores across 17 participants following Sativex® administration

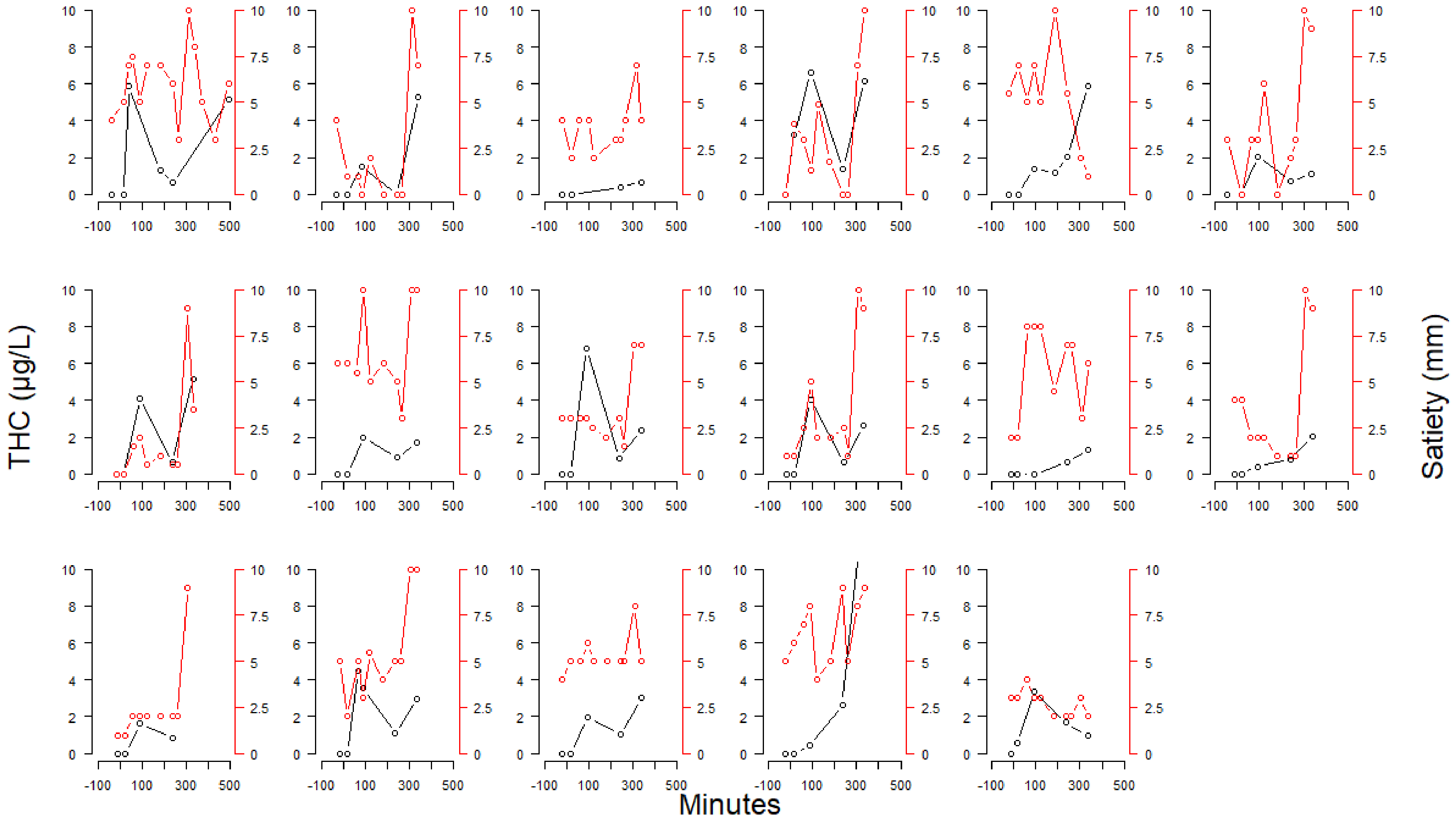

Figure S10. Time courses of individual THC concentrations and combined appetite scores across 17 participants following Sativex® administration

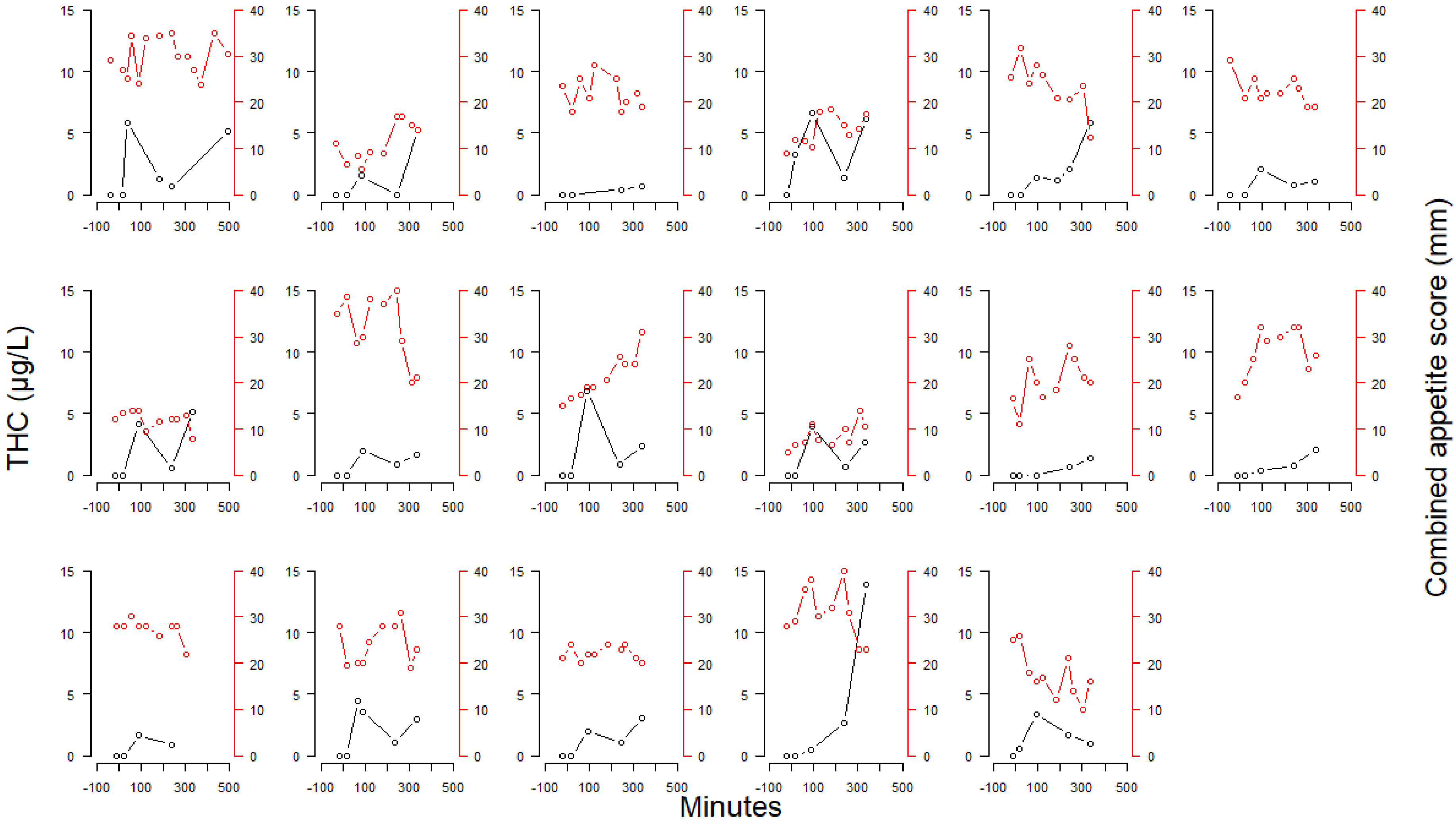

Figure S11. Time courses of individual 11-OH-THC and postprandial GLP-1 concentration across 17 participants following Sativex® administration

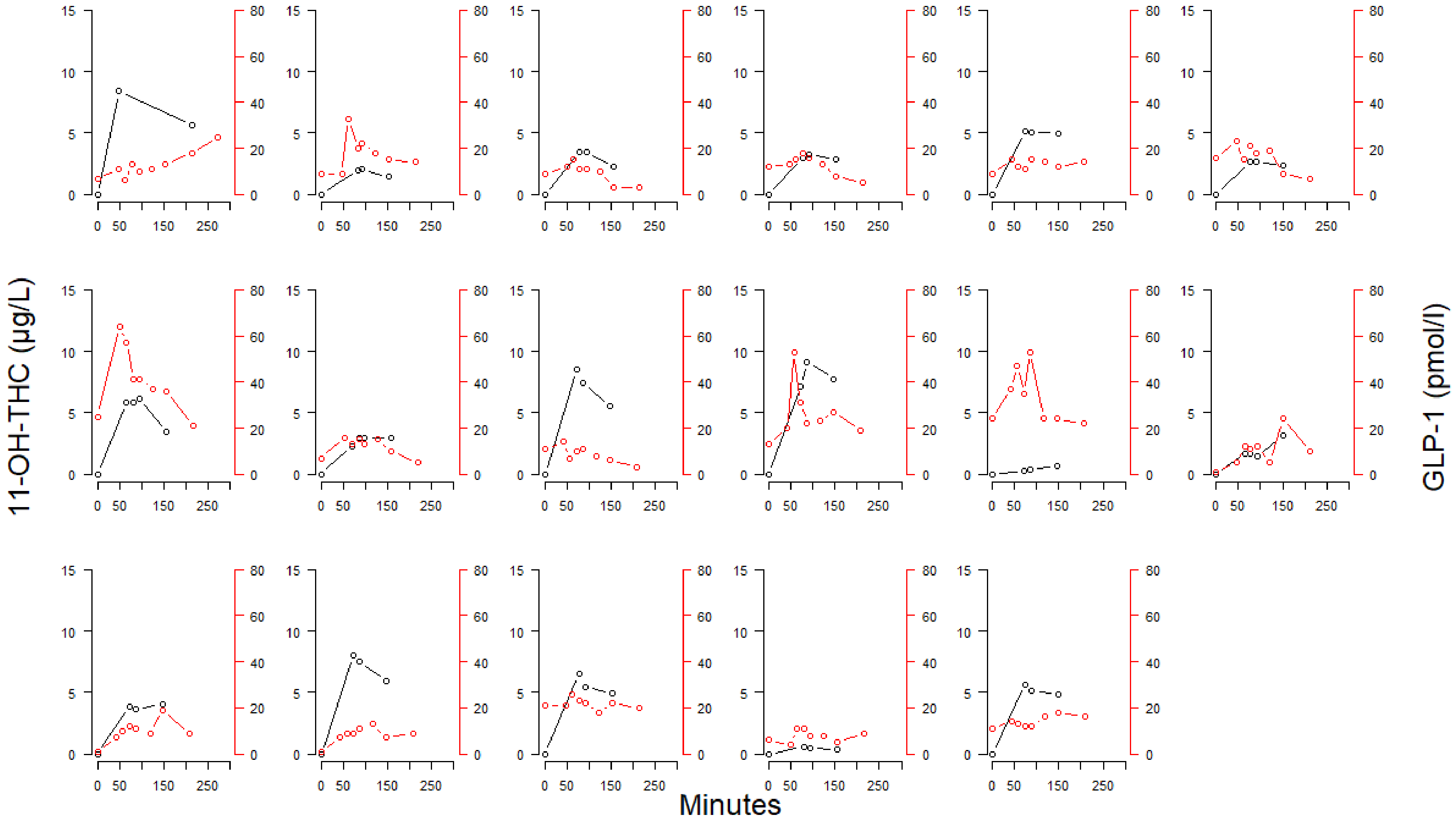

Figure S12. Time courses of individual 11-OH-THC and postprandial total ghrelin concentration across 17 participants following Sativex® administration

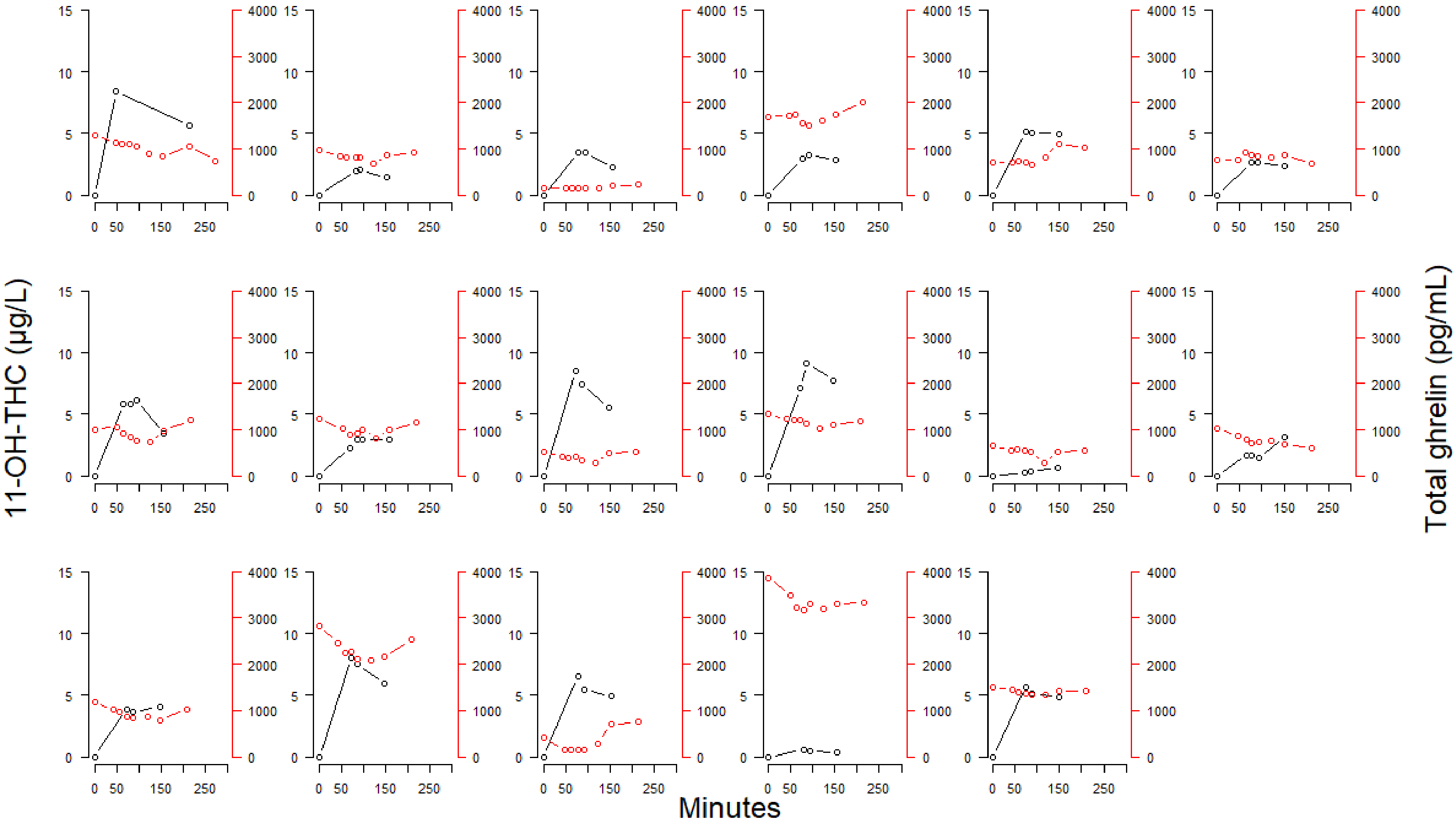

Figure S13. Time courses of individual 11-OH-THC concentrations and desire to eat scores across 17 participants following Sativex® administration

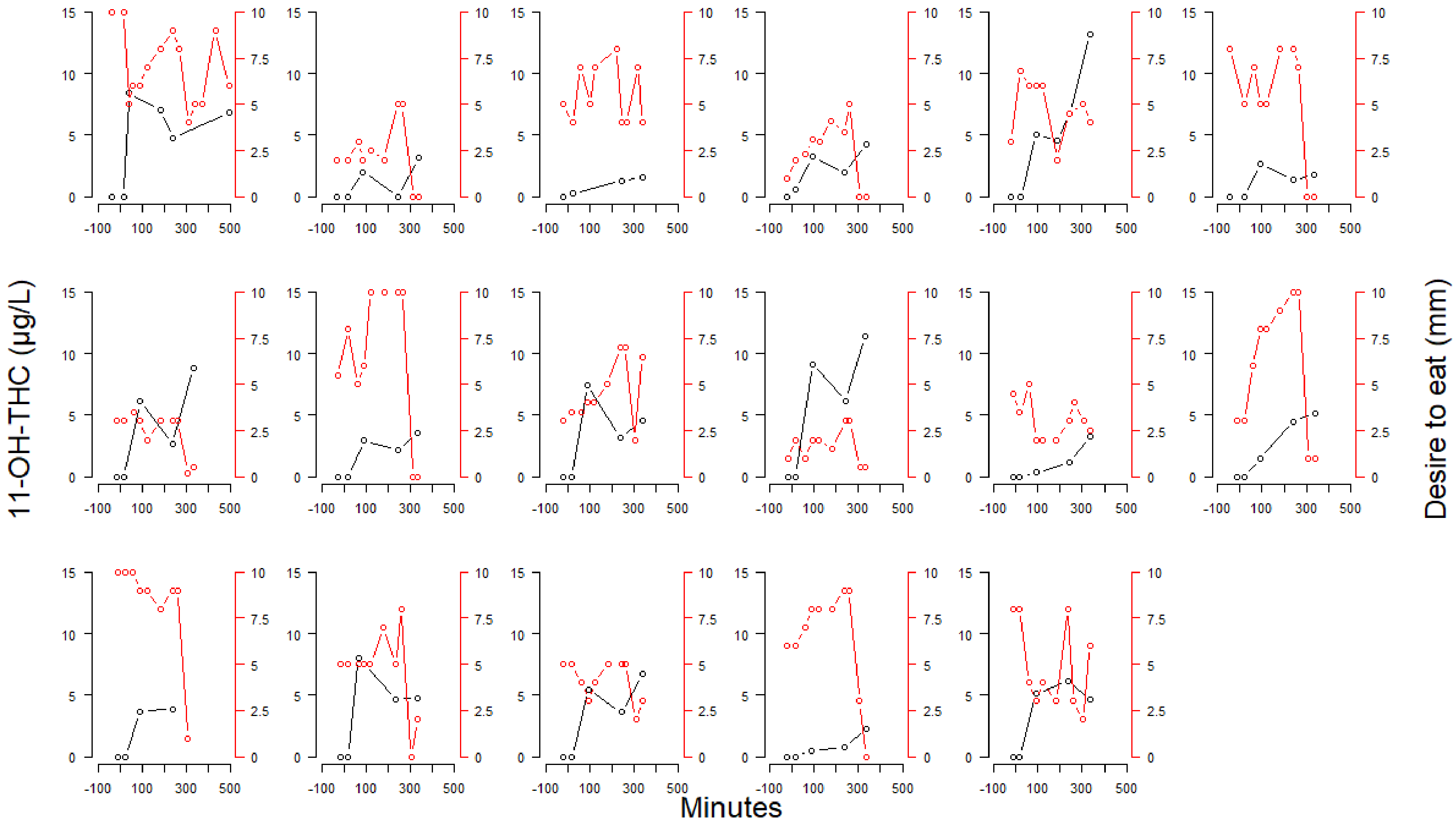

Figure S14. Time courses of individual 11-OH-THC concentrations and future food intake scores across 17 participants following Sativex® administration

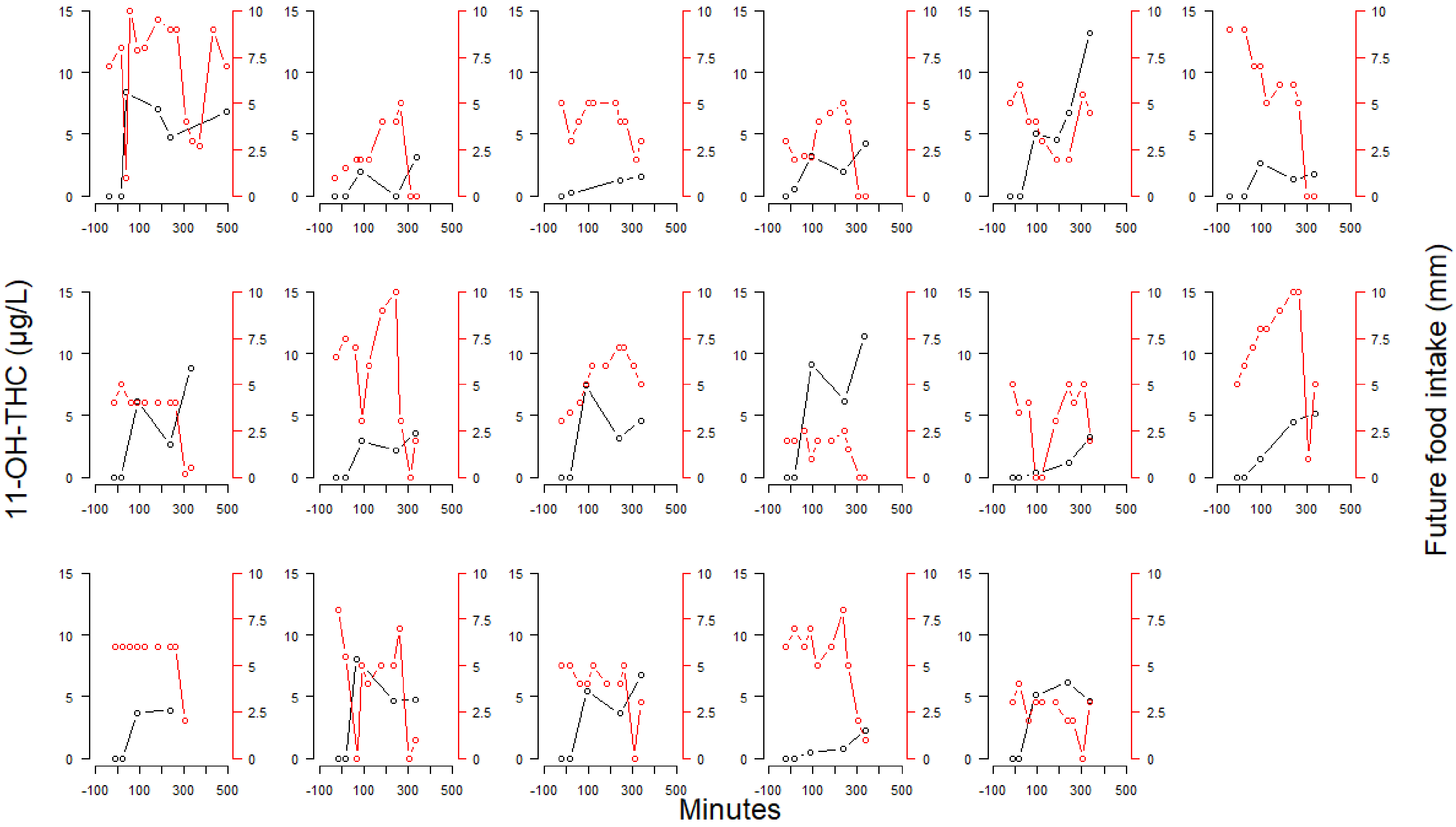

Figure S15. Time courses of individual 11-OH-THC concentrations and fullness scores across 17 participants following Sativex® administration

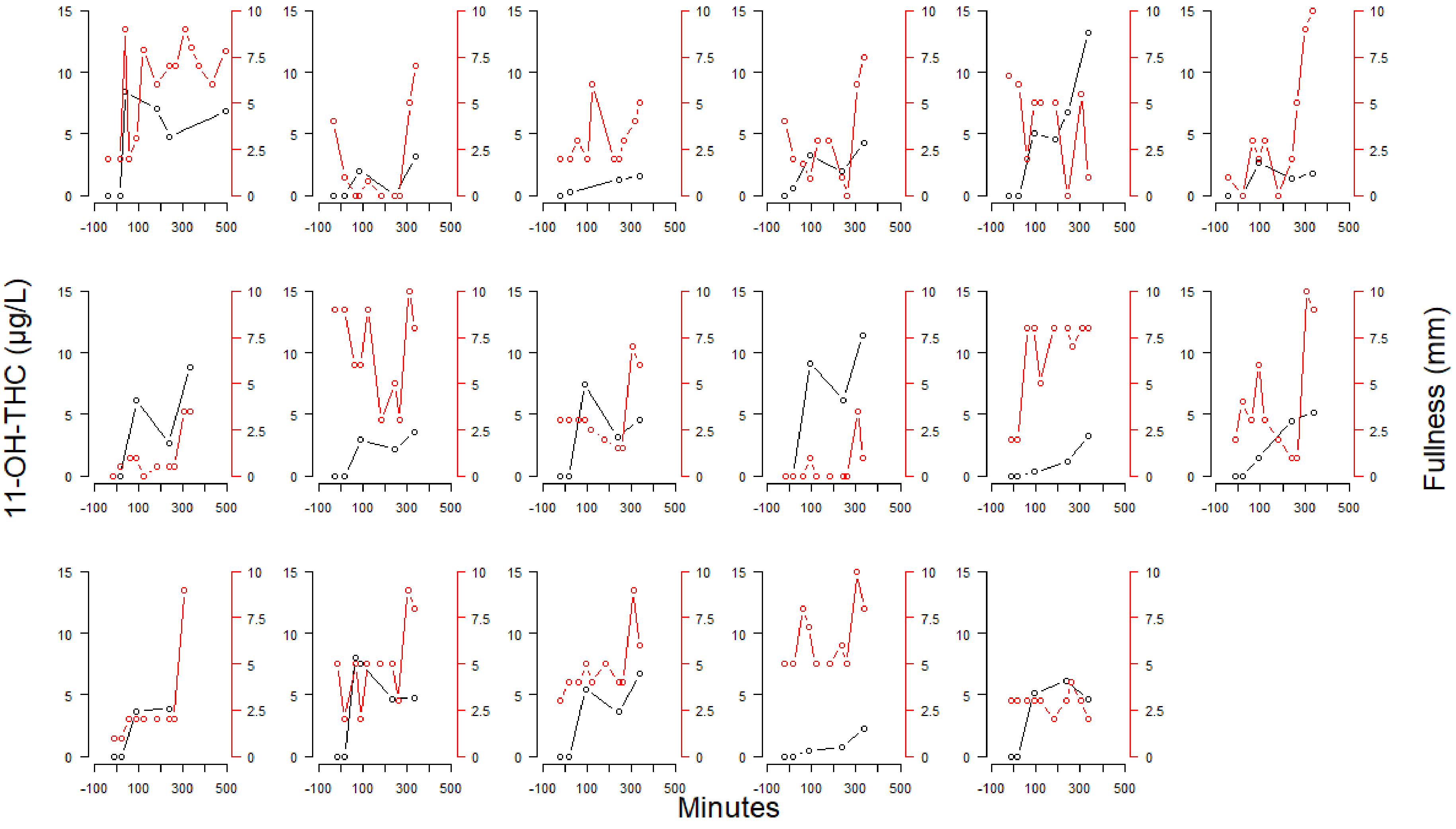

Figure S16. Time courses of individual 11-OH-THC concentrations and hunger scores across 17 participants following Sativex® administration

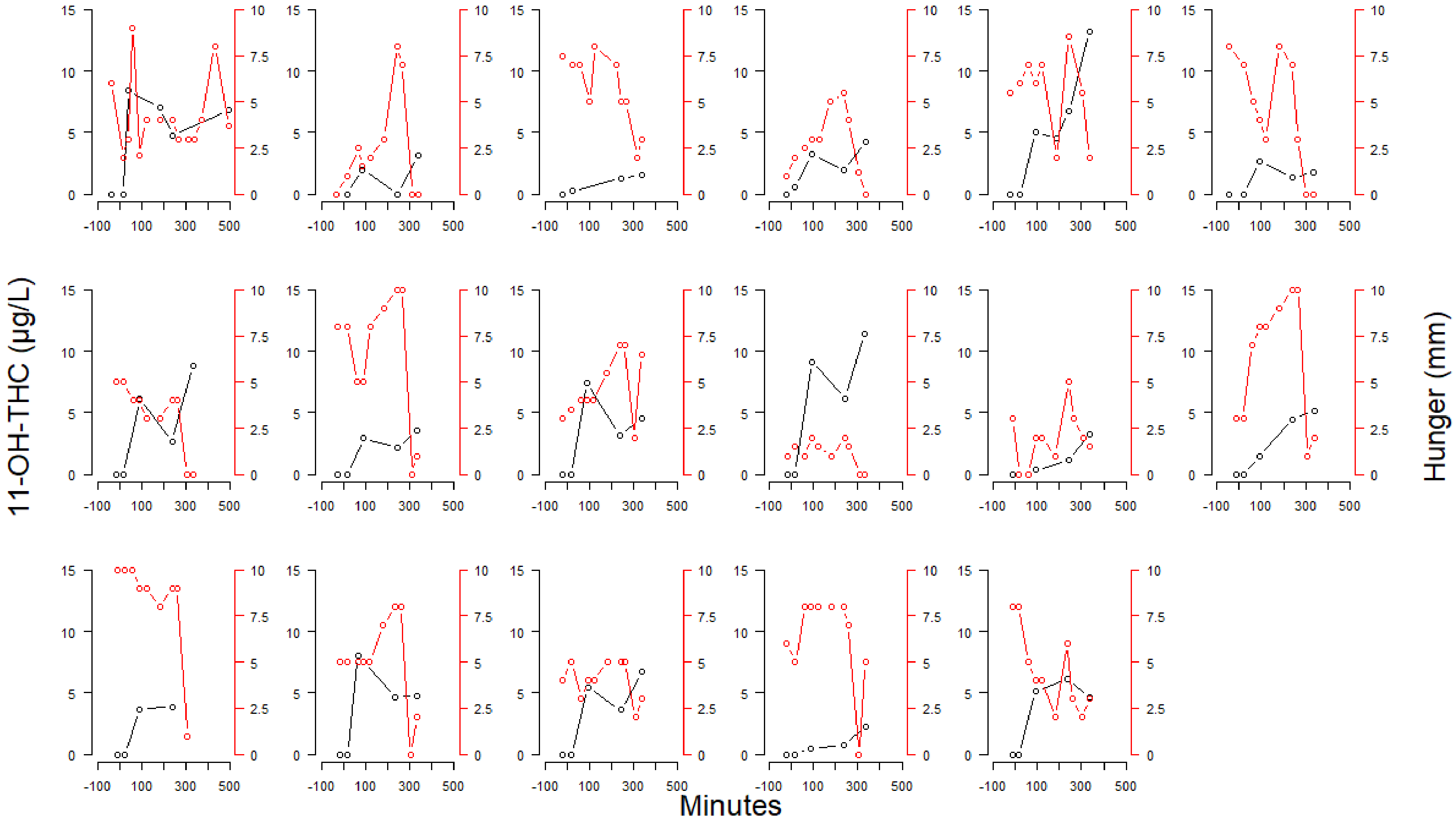

Figure S17. Time courses of individual 11-OH-THC concentrations and satiety scores across 17 participants following Sativex® administration

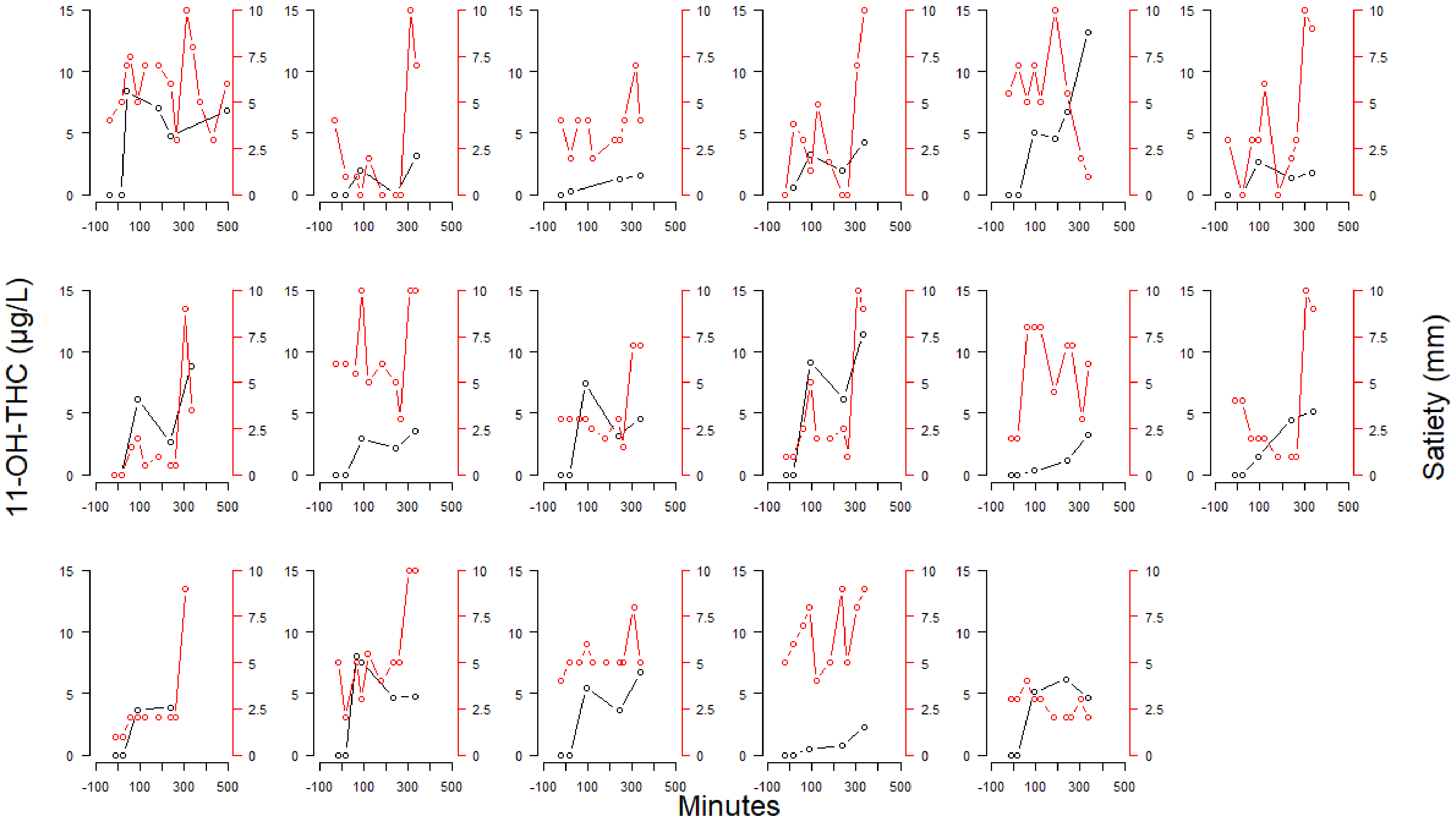

Figure S18. Time courses of individual 11-OH-THC concentrations and combined appetite scores across 17 participants following Sativex® administration

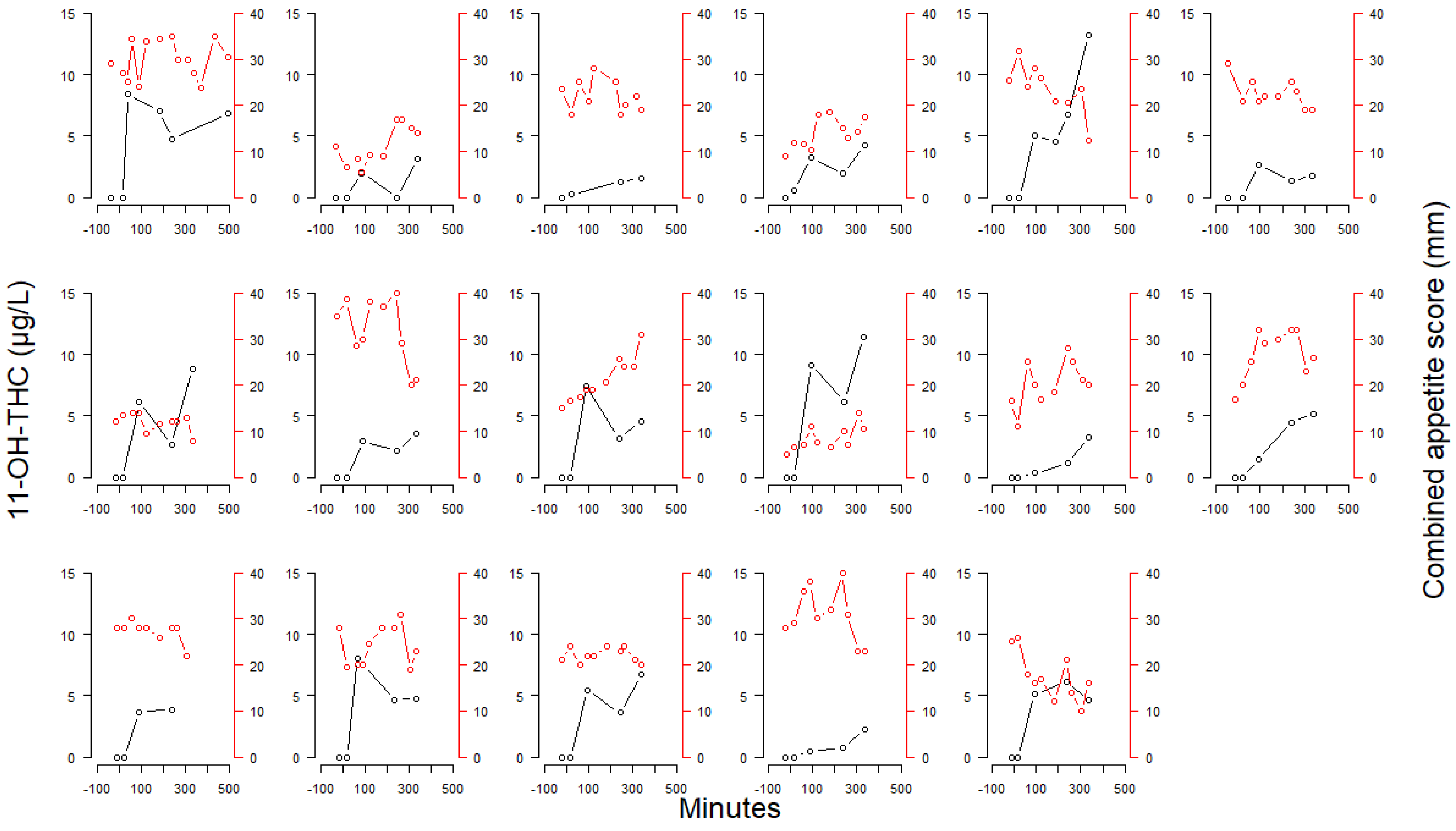

Figure S19. Time courses of individual THC-COOH and postprandial GLP-1 concentrations across 17 participants following Sativex® administration

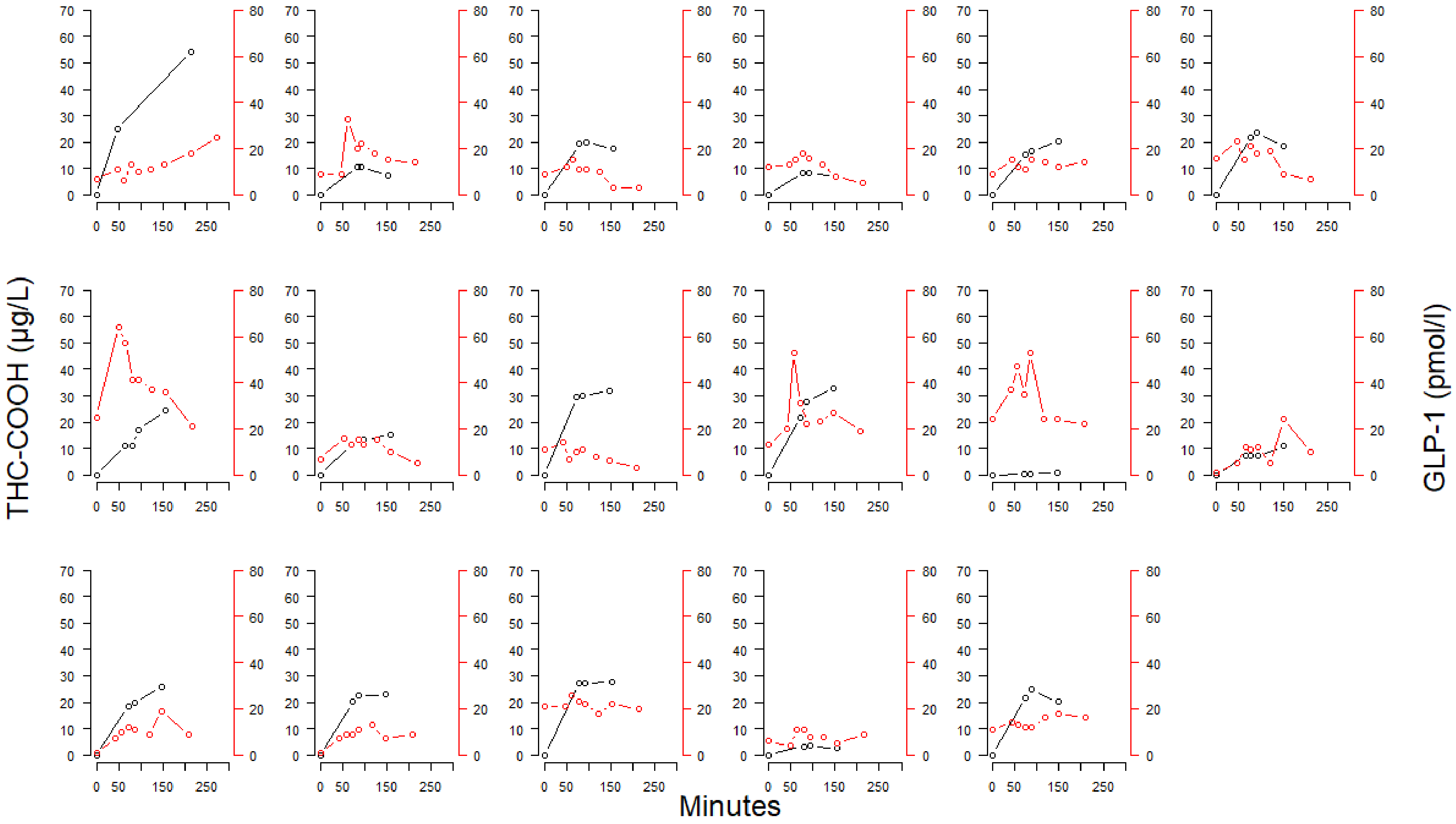

Figure S20. Time courses of individual THC-COOH and postprandial total ghrelin concentrations across 17 participants following Sativex® administration

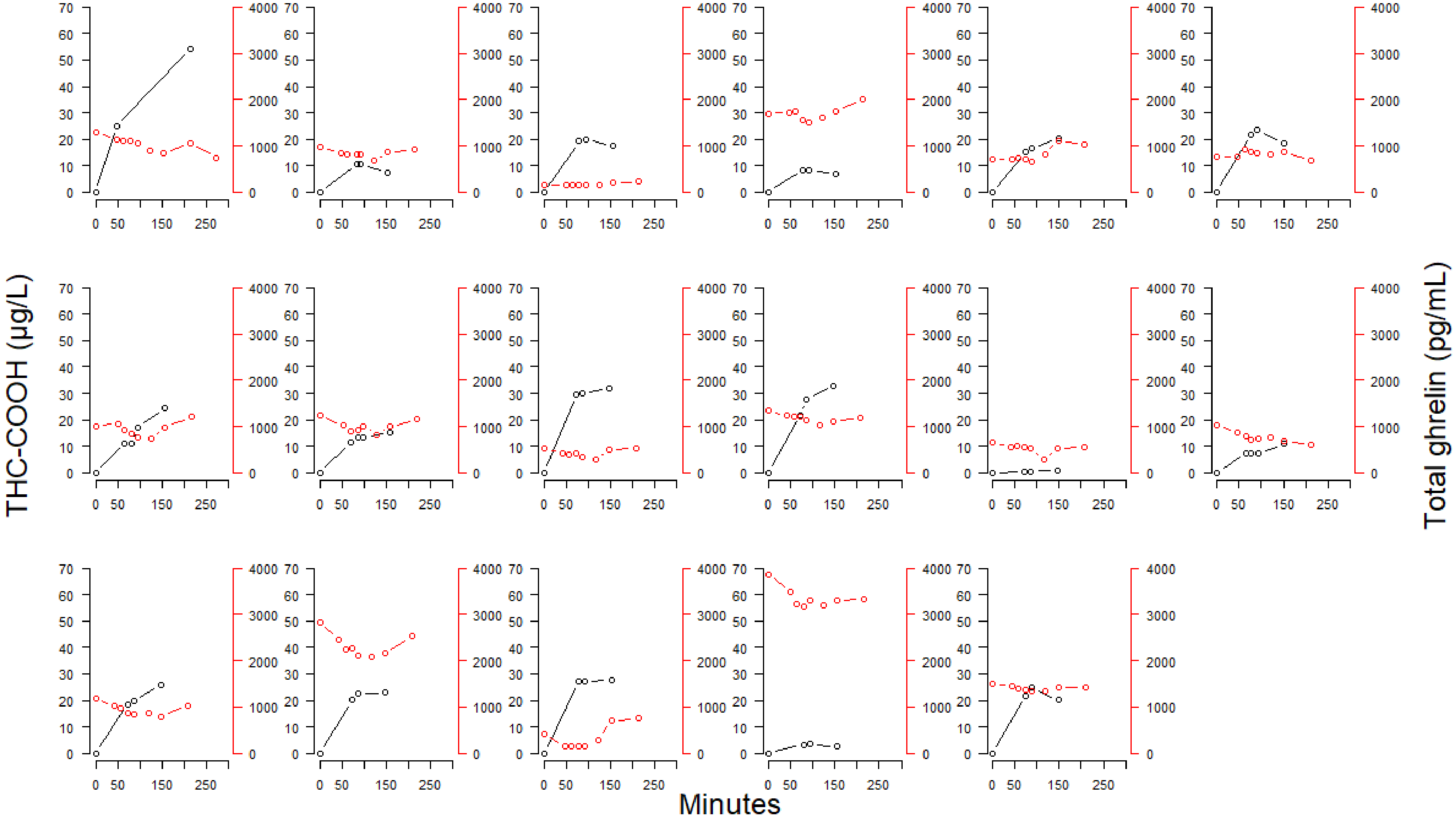

Figure S21. Time courses of individual THC-COOH concentrations and desire to eat scores across 17 participants following Sativex® administration

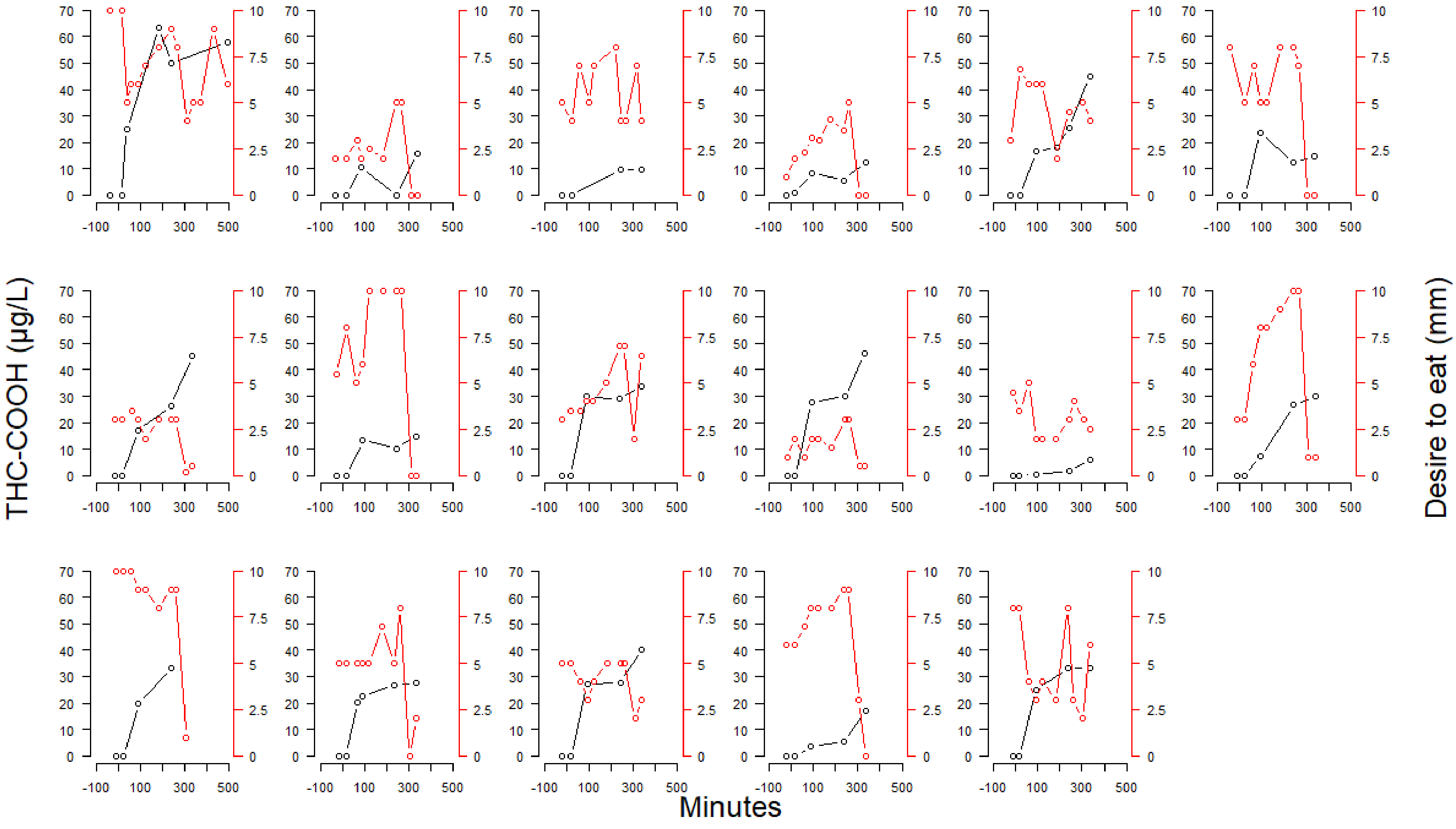

Figure S22. Time courses of individual THC-COOH concentrations and future food intake scores across 17 participants following Sativex® administration

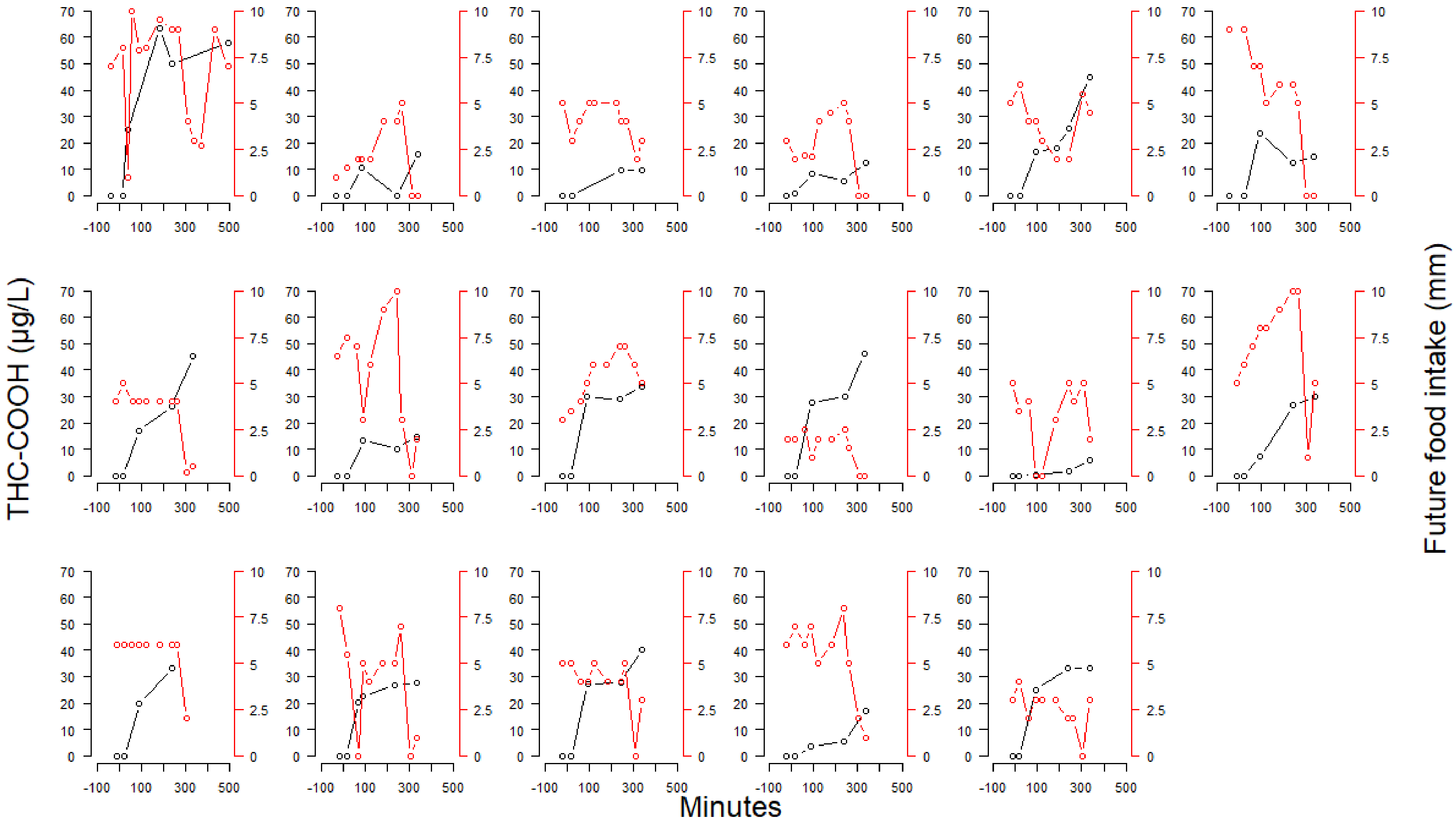

Figure S23. Time courses of individual THC-COOH concentrations and fullness scores across 17 participants following Sativex® administration

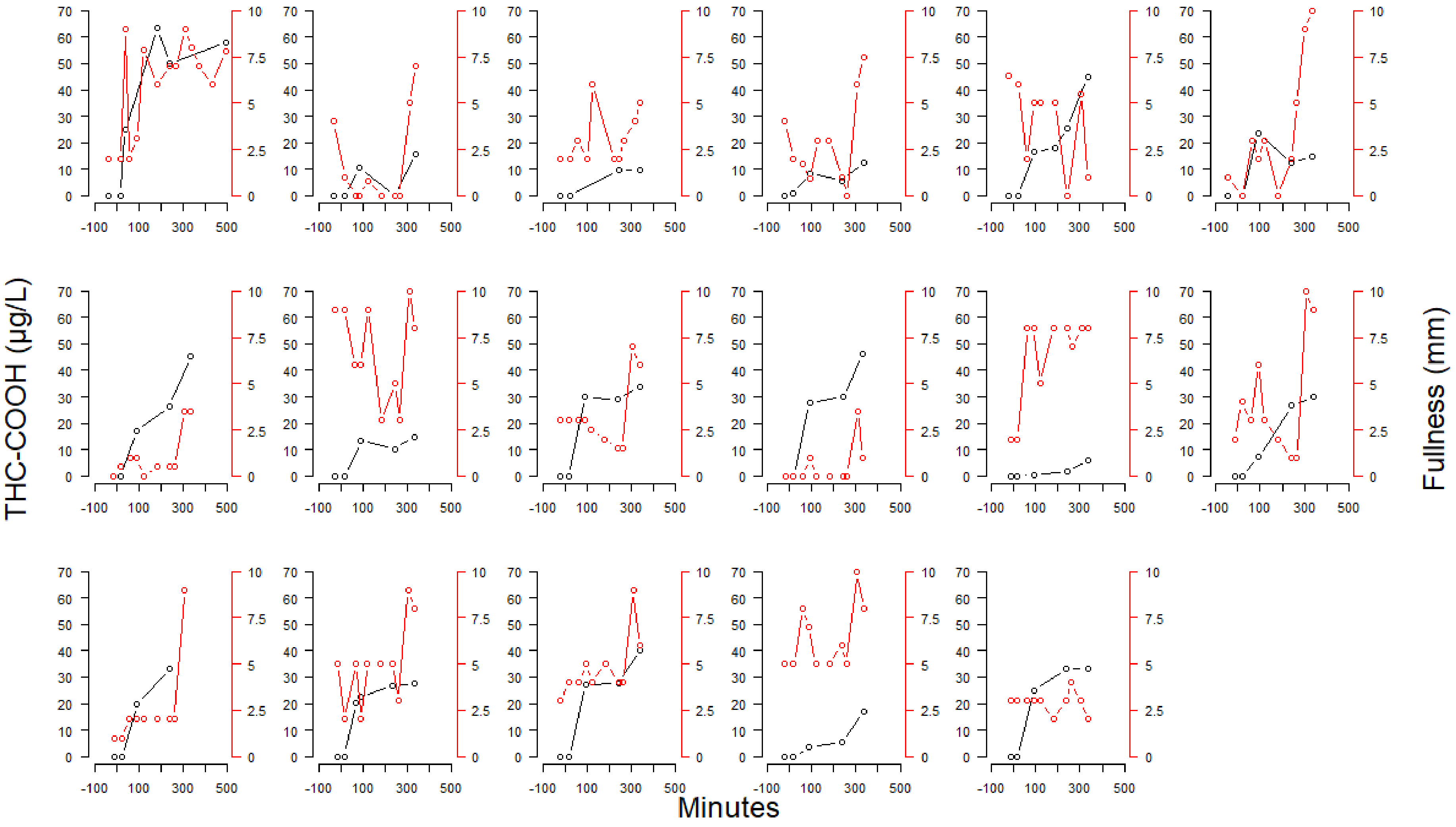

Figure S24. Time courses of individual THC-COOH concentrations and hunger scores across 17 participants following Sativex® administration

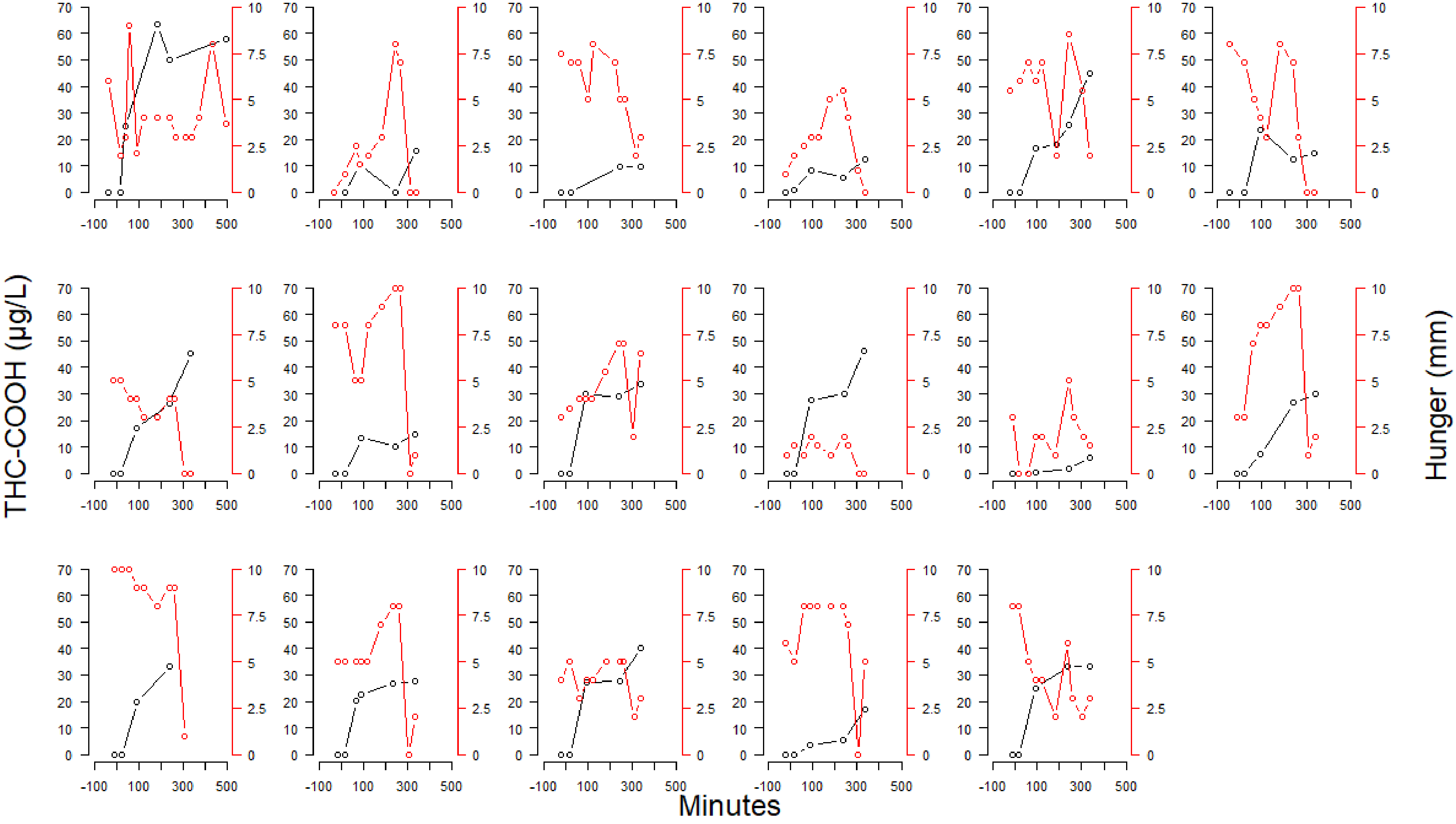

Figure S25. Time courses of individual THC-COOH concentrations and satiety scores across 17 participants following Sativex® administration

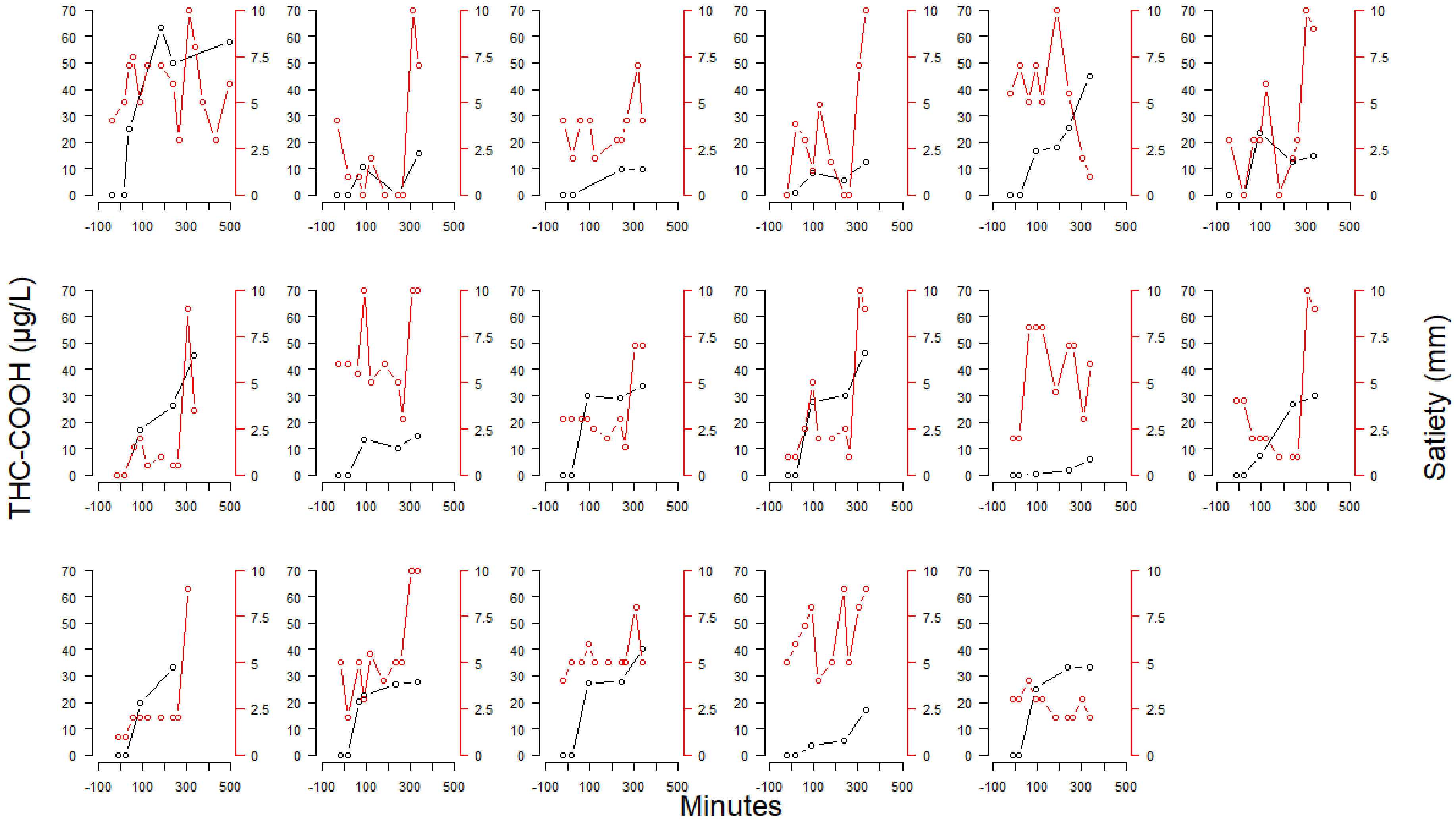

Figure S26. Time courses of individual THC-COOH concentrations and combined appetite scores across 17 participants following Sativex® administration

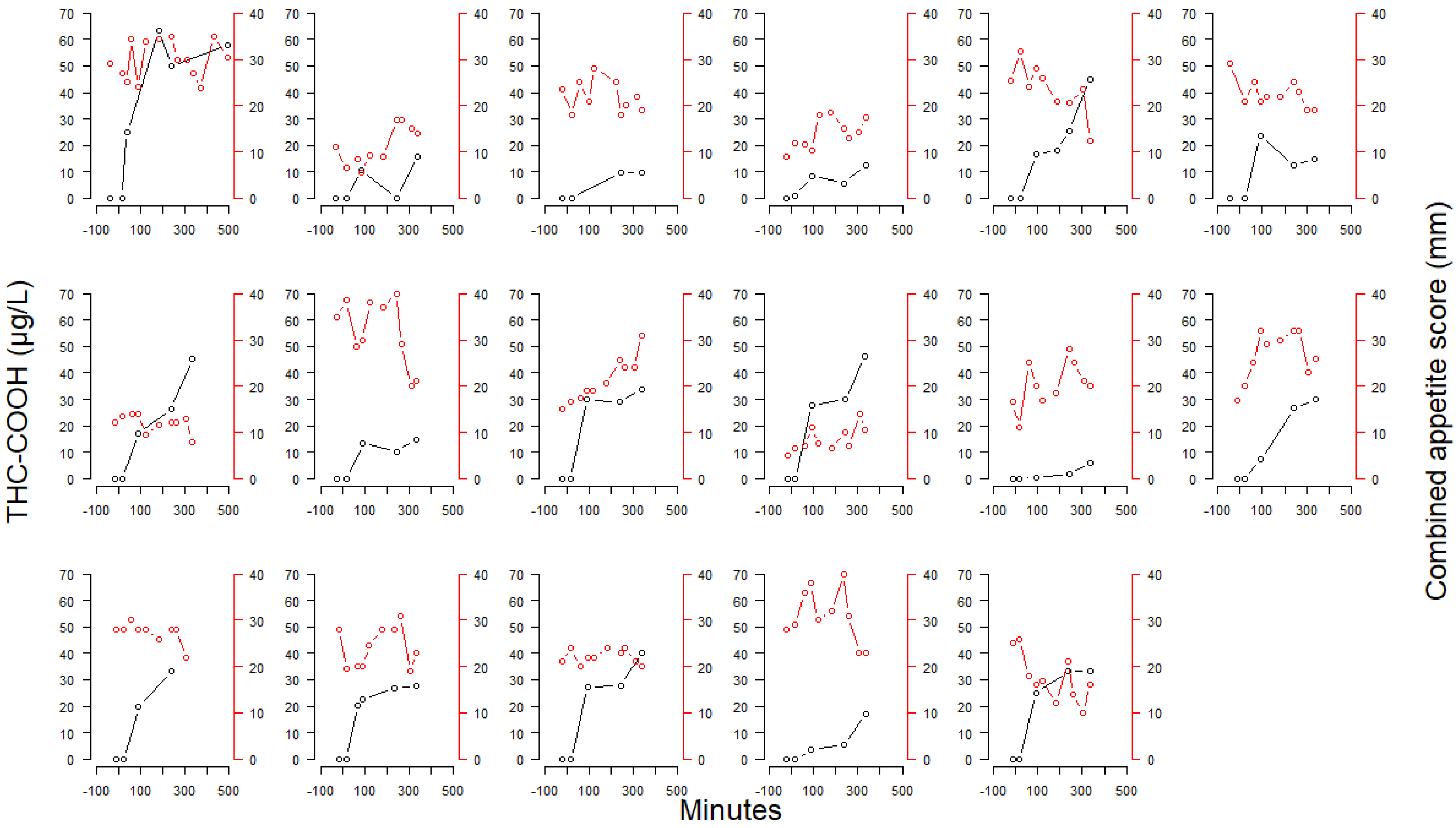

Figure S27. Time-dependent changes for THC, 11-OH-THC, and THC-COOH

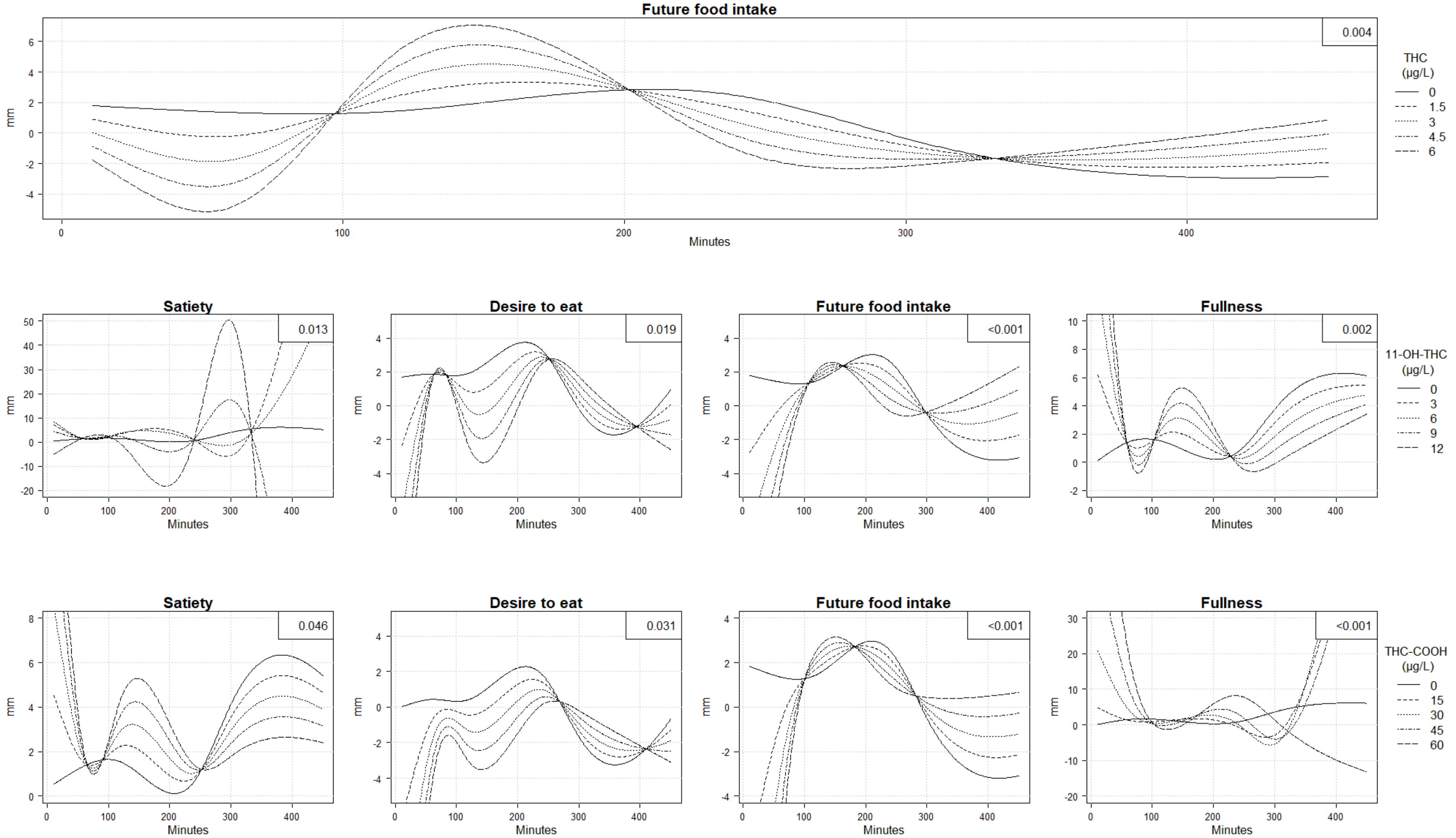

Supplement: Supplementary file 1 [file nutrients-18-02274-s001.zip › nutrients-4399549-supplementary.pdf]
